# Stunting is associated with persistent and transferable alterations in the gut microbiome

**DOI:** 10.1186/s13099-025-00723-2

**Published:** 2025-06-25

**Authors:** Joshua O. Amimo, C. N. Kunyanga, S. A. Raev, M. Kick, H. Micheal, L. J. Saif, Anastasia N. Vlasova

**Affiliations:** 1 Center for Food Animal Health, Department of Animal Sciences, College of Food, Agricultural and Environmental Sciences, The Ohio State University, 1680 Madison Avenue, Wooster, OH 44691 USA; 2 Department of Food Science, Nutrition and Technology, Faculty of Agriculture, University of Nairobi, P.O Box 29053, Nairobi, 00625 Kenya

**Keywords:** (Child gut microbiota, Fecal microbiota transplantation, Gnotobiotic pig model, Malnutrition/stunting, Microbial colonization, Gut microbiota functions)

## Abstract

As robust animal models to study the pathophysiology of stunting are absent, we have comparatively characterized the gut microbiota of malnourished/stunted vs. clinically healthy/normal Kenyan toddlers (12–24 months old) and established a gnotobiotic (Gn) pig fecal transplant model to gain understanding of microbial community structure associated with stunting. As expected, the bacterial composition between the two toddler groups was distinct: *Actinobacteria* was most prevalent in healthy toddlers, whereas *Proteobacteria* dominated in stunted toddlers. Although the diversity indices showed no significant differences, unique bacterial genera were found in each toddler group: three genera unique to stunted toddlers and ten unique to healthy toddlers, with eight genera shared between the groups. We observed a higher number of enriched bacterial virulence genes in healthy vs. stunted toddlers suggesting that the microbiome plasticity and functional characteristics of the healthy toddlers allow for the pathogen/pathobiont control. In contrast, we noted the presence of more genes associated with antimicrobial-resistance (AMR) bacteria in stunted toddlers, possibly due to early-life antibiotic treatments. Of interest, functional analysis showed that CAZymes associated with carbohydrate biosynthesis, and a few metabolic pathways related to protein/amino acid, carbohydrate and fat catabolism were enriched in stunted toddlers. In contrast carbohydrate degradation CAZymes and numerous anabolic pathways were prevalent in healthy toddlers. These patterns were also evident in the Gn pigs transplanted with stunted/healthy human fecal microbiota (HFM). Overall, our findings suggest that the microbiota transplanted Gn pigs represent a valuable model for studying the infant microbial community structure and the impacts of stunting on the child gut microbiota. Additionally, this is the first study to demonstrate that the healthy vs. stunted microbiota composition and function remained different in the Gn pigs throughout the study. This information and the Gn pig model are vital for developing and testing targeted interventions for malnourished/stunted populations, consequently advancing microbiome-based diagnosis and personalized medicine.

**Supplementary Information:**

The online version contains supplementary material available at 10.1186/s13099-025-00723-2.

## Introduction

Malnutrition is a leading cause of morbidity and mortality globally, especially in low- and middle-income countries [1]; hence, understanding gut microbiome community structure offers insights into targeted interventions to address this burden. Studying the malnutrition-associated microbiome and long-term health sequelae (such as stunting) is essential due to its profound implications for understanding disease mechanisms, improving public health interventions, and developing innovative therapies. Malnutrition disrupts the gut microbiome, leading to reduced microbial diversity and altered metabolic activity, impairing nutrient absorption and exacerbating malnutrition [2]. Moreover, a disrupted microbiome can weaken immune responses, increasing susceptibility to infections. The microbiome plays a vital role in synthesizing vitamins and short-chain fatty acids essential for gut health and systemic metabolism; thus, understanding these processes can improve nutritional supplementation strategies [3, 4]. Monitoring microbiome changes in malnutrition and stunting can serve as an early warning system for public health interventions, thus underscoring the importance of considering the gut microbiota in strategies to combat malnutrition and its long-term health consequences [5, 6]. Besides, studying malnutrition-associated microbiome changes, both in human and in animal models, enhances our understanding of host-microbe interactions, metabolic pathways, and the microbiome’s role in maintaining health, thereby advancing basic science.

The gut microbiome influences human health through various mechanisms, including modulating gut permeability, which in turn affects nutrient absorption and waste excretion [7]. It also plays a role in inflammation by activating immune cells and producing both proinflammatory and anti-inflammatory molecules. Furthermore, it influences neurotransmitter synthesis, hormone regulation, and the production of bacterial metabolites such as short-chain fatty acids, secondary bile acids, and branched-chain amino acids [8, 9]. Studies have shown that there is high taxonomic diversity, high microbial gene richness and stable core microbiota in a healthy gut microbiota [10]. Therefore, effective microbial interventions and therapies for chronic diseases require a comprehensive understanding of microbiome assembly and its effects on host metabolism and health. Research indicates that normal gut microbiota contributes specific functions in nutrient metabolism, xenobiotic and drug metabolism, maintaining gut mucosal integrity, immunomodulation, and pathogen protection [8, 11]. Microbiota composition varies within individual body sites, with six major bacterial phyla present in the human gut—*Firmicutes*,* Bacteroidetes*,* Actinobacteria*,* Proteobacteria*,* Fusobacteria*, and *Verrucomicrobia*, whereby *Firmicutes* and *Bacteroidetes* are predominant [7, 12]. Fungi, viruses, archaea, and phages are also components of the gut microbiota ecosystem [13, 14]. To manipulate gut microbiota effectively in therapeutic contexts, understanding its functions and the role in gastrointestinal and other diseases is essential. The microbiota influences energy and nutrient extraction from food through diverse metabolic genes providing unique enzymes and pathways [15–17]. It is also crucial for the biosynthesis of bioactive molecules such as vitamins, amino acids, and lipids [18, 19]. Finally, the microbiota not only produces antimicrobial substances to protect against pathogens, but it also supports intestinal mucosa and immune system development [7].

Exploring the connections between human phenotypes and the microbiome is challenging due to confounding factors such as genetic diversity, environmental influences, and dietary variations, especially in low-resource settings. Therefore, animal models that allow microbiome colonization and manipulation in a controlled environment are necessary to understand the impact on the host. Metagenomic studies indicate that infections can alter microbial community composition and function, affecting immunity, nutrient bioavailability, metabolism, and gut barrier function [20–23]. Despite challenges in comprehensive characterization of the microbiome’s role in human health, recent use of germ-free or gnotobiotic (Gn) animal models, including mice, zebrafish, and swine, has been invaluable in exploring microbial interactions [24–32]. These models offer realistic environments for studying microbe-microbe and microbe-host interactions, aiding our understanding of their roles in host development and maturation, especially in the immune and endocrine systems [7, 33]. Additionally, recent advances in next-generation sequencing and metagenomics have facilitated detailed microbial community characterization [34–36], highlighting the importance of understanding microbial impact on human health and identifying metabolic pathway functions. This understanding supports research in personalized nutrition and microbiome-targeted therapies [14]. We examined the differences in the fecal microbiota diversity and functions between healthy and malnourished or stunted Kenyan toddlers from low-resource settings and assessed profiles of these microbiota in fecal transplant gnotobiotic (Gn) pig models. This model represents a physiologically relevant system with closer anatomical and immune resemblance to humans than rodents—to investigate the persistence and transferability of gut microbiota from malnourished children. The Gn piglet model is especially valuable due to its physiological similarity to the human gastrointestinal tract and immune system, offering a more translational model compared to rodents. Although the sample size is limited, the model offers insight into microbial colonization patterns. We hypothesized that transplanting human toddler fecal microbiota into these Gn pigs would result in a similar gut microbiota composition. The study involved orally inoculating neonatal Gn piglets with human toddler fecal microbiota and maintaining them on a sterile bovine milk diet for 23 days. Analysis of bacterial DNA from the toddler fecal inoculum and piglet large intestinal contents through deep sequencing validated the transplantation success, offering insights into developing improved human gut microbiome models. Compared to previous studies that mostly used low sensitive methods (16 rRNA sequencing), our approach of whole genome sequencing allows for a more accurate identification and quantification of bacterial species as well as comparison of the functional features. Additionally, our study is the first one to establish a physiologically relevant and robust animal model that recapitulates the compositional and functional characteristics of the stunting-associated microbiome and demonstrates stability of the microbial communities.

## Materials and methods

### Human (toddlers) fecal microbiota from low resource settings

In collaborative research between the University of Nairobi (UoN) and Ministry of Health, Kenya, and with parental consent, fecal samples were collected by trained clinicians at Kenyatta National Hospital (National referral hospital). The sample collection for this study was approved by the Kenyatta National Hospital– UoN Ethics and Research Committee (#P708/10/2016). The inclusion criteria were less than two years old, vaginally-born children/toddlers (clinically healthy/normal, and/or malnourished/stunted), and exclusively breastfed. However, dietary history collected during the sampling indicated that 0–6 months old toddlers were exclusively breast-fed, while 6–12 months toddlers (fecals sample donors) were partially breastfed and provided solid foods. Complementary diet consisted of pureed/blended food with Irish potatoes, green bananas, green leafy vegetables and cabbage; minced meat gravy/soup; tea with more milk; special porridge with milk or eggs for high protein, blended soybean and maize porridge. Stunted status was defined as low height-for-age based on the World Health Organization (WHO) definition, where a child was considered stunted if their height-for-age Z-score is more than two standard deviations below the WHO Child Growth Standards median. The exclusion criteria were toddlers with: (i) any non-gastrointestinal infections (Hepatitis B or C, human immunodeficiency virus (HIV), arboviral fevers, influenza, common colds); (ii) acute gastrointestinal infections (rotaviruses, noroviruses, coronaviruses, caliciviruses, Hepatitis A, non-typhoidal salmonella, enterotoxigenic *E. coli*, Shigella, Clostridium perfringens); (iii) cesarean section births; and (iv) formula-fed toddlers that can affect the microbiome composition and the study results. Fecal samples (~ 30 g depending on availability) were collected in sterile vials (plastic 50-mL vial) immediately after defecation, placed in reduced media [phosphate buffer solution containing 0.05% (vol/vol) cysteine and 30% sterile glycerol] [37], refrigerated and transported to the Microbiology Lab, Department of Food Science, Nutrition and Technology, Faculty of Agriculture, University of Nairobi. The fecal samples were stored directly in the reduced medium at − 80 °C without removal to minimize oxygen exposure. A list of all samples with toddlers’ data were kept at the University of Nairobi. Coded, de-identified fecal samples were shipped on dry ice to the Center for Food Animal Health, The Ohio State University for microbiome analysis and colonization dynamics study in a fecal transplant gnotobiotic (Gn) pig model. Randomly selected (based on nutritional status, absence of acute illness, and no antibiotic use in the preceding month) fecal samples from clinically healthy (*n* = 3) and malnourished/stunted (*n* = 3) toddlers aged 12–24 months were used for microbiota analysis. Dietary data of these selected toddlers indicated mixed feeding practices, including partial breastfeeding and the introduction of complementary foods. Of the randomly selected fecal samples, one representative sample from each toddler group was randomly selected for fecal microbiota transplantation in Gn pigs. A single-donor inoculum was used to provide a more consistent and controlled microbial composition and to minimize inter-individual variability. We were cognizant of the fact that mixed donor inocula could lead to microbial competition, resulting in dominance by certain strains or unpredictable community structures. Besides this, multiple donors could dilute or obscure key microbial signals relevant to our research question.

### Animal experiments

The animal experiments for this study were approved by The Ohio State University Institutional Animal Care and Use Committee. Piglets were derived from near-term conventional sows by hysterectomy and maintained in sterile isolators as Gn piglets. Neonatal Gn pigs (*n* = 19) were randomly assigned to one of 2 groups and housed in 5 isolators of 4 piglets each: (1) Normal toddler fecal microbiota inoculated (normal Gn-piglets, *n* = 11) and (2) stunted toddler fecal microbiota inoculated (stunted Gn-piglets, *n* = 8). All piglets were fed 100% ultra-high temperature pasteurized bovine milk (Parmalat) that met the National Research Council Animal Care Committee’s guidelines for calories, fat, protein, and carbohydrates in suckling pigs. At 3 days of age, all pigs underwent sterility tests to confirm that they are free from bacterial and fungal contamination before the start of the experiment. At the same age piglets were injected with Iron and fed sterile sow serum orally to prevent septicemia. Sterile sow serum was administered daily for 5 consecutive days. Piglets were orally inoculated with 2 ml of diluted (1:50) toddler fecal material stock at 6 days of age (post bacterial transplantation day, PTD0). Piglets blood (for blood cell count), and weight were analyzed/recorded before fecal inoculation at PTD0, and at PTD7, PTD13 and PTD23. A subset of piglets (*n* = 4 per group) was randomly selected and euthanized at PTD7 to assess the microbial community structure post-transplantation at this age. The remaining piglets were euthanized at PTD23, and the microbiota composition was evaluated. Large intestinal contents were collected to evaluate the microbiota diversity and functions and compared with the microbiota present in the toddler fecal inoculum. The Gn pigs were monitored daily for symptoms and clinical signs of disease post transplantation.

### DNA extraction from large intestinal contents and next generation sequencing

Samples (toddler feces and large intestinal contents of Gn pigs) were thawed at room temperature (~ 23 °C) and thoroughly mixed by vortex at maximum speed for 30 s. Total DNA was extracted from the samples using QIAamp DNA Stool Mini Kit (Qiagen, Valencia, CA, USA), following the manufacturer’s instructions with minor modifications. Modifications included doubling the amount of starting fecal material, RNase A and proteinase K treatment. Total DNA eluded was quantified using nanodrop spectrophotometer (ThermoScientific, USA) and submitted on dry ice for sequencing to COSMOID Inc. (https://www.cosmosid.com). At COSMOID, DNA samples were quantified using Qubit 4 fluorometer and Qubit™ dsDNA HS Assay Kit (Thermofisher Scientific) to check quality and quantity after shipment.

DNA Library Preparation and Sequencing Methods: DNA libraries were prepared using the Nextera XT DNA Library Preparation Kit (Illumina) and IDT Unique Dual Indexes with total DNA input of 1ng. Genomic DNA was fragmented using a proportional amount of Illumina Nextera XT fragmentation enzyme. Unique dual indexes were added to each sample followed by 12 cycles of PCR to construct libraries. DNA libraries were purified using AMpure magnetic Beads (Beckman Coulter) and eluted in QIAGEN EB buffer. DNA libraries were quantified using Qubit 4 fluorometer and Qubit dsDNA HS Assay Kit. Libraries were then sequenced on Illumina NovaSeq platform 2 × 150 bp.

### Data analysis

Bioinformatics Analysis via CosmosID-HUB Methods *(*www.cosmosid.com*)*: The system utilizes a high-performance data-mining k-mer algorithm that rapidly disambiguates millions of short sequence reads into the discrete genomes engendering the particular sequences. The pipeline has two separable comparators: the first consists of a pre-computation phase for reference databases and the second is a per-sample computation. The input to the pre-computation phase are databases of reference genomes, virulence markers and antimicrobial resistance markers that are continuously curated by CosmosID scientists. The output of the pre-computational phase is a phylogeny tree of microbes, together with sets of variable length k-mer fingerprints (biomarkers) uniquely associated with distinct branches and leaves of the tree. The second per-sample computational phase searches the hundreds of millions of short sequences reads, or alternatively contigs from draft de novo assemblies, against the fingerprint sets. This query enables the sensitive yet highly precise detection and taxonomic classification of microbial NGS reads. The resulting statistics are analyzed to return the fine-grain taxonomic and relative abundance estimates for the microbial NGS datasets. To exclude false positive identifications, the results are filtered using a filtering threshold derived based on internal statistical scores that are determined by analyzing a large number of diverse metagenomes. The same approach was applied to enable the sensitive and accurate detection of genetic markers for virulence and for resistance to antibiotics.

Functional Analysis via CosmosID-HUB: Initial QC, adapter trimming and preprocessing of metagenomic sequencing reads are done using BBduk [38]. The quality-controlled reads are then subjected to a translated search against a comprehensive and non-redundant protein sequence database, UniRef 90. The UniRef90 database, provided by UniProt [39], represents a clustering of all non-redundant protein sequences in UniProt, such that each sequence in a cluster aligns with 90% identity and 80% coverage of the longest sequence in the cluster. The mapping of metagenomic reads to gene sequences are weighted by mapping quality, coverage and gene sequence length to estimate community wide weighted gene family abundances as described by Franzosa et al. [40]. Gene families are then annotated to MetaCyc reactions (Metabolic Enzymes) to reconstruct and quantify MetaCyc [41] metabolic pathways in the community as described by Franzosa et al. [40]. Furthermore, the UniRef_90 gene families are also regrouped to Enzyme Commission Enzymes, Pfam protein domains, CAZy enzymes and GO Terms in order to get an exhaustive overview of gene functions in the community. Lastly, to facilitate comparisons across multiple samples with different sequencing depths, the abundance values are normalized using Totalsum scaling (TSS) normalization to produce “Copies per million” (analogous to TPMs in RNASeq) units.

## Results

In this study we evaluated the gut microbiota diversity and functions of four (4) groups; (1) clinically healthy toddlers (Normal_H, *n* = 3); (2) malnourished/stunted toddlers (Stunted_H, *n* = 3); (3) Gn piglets transplanted with human fecal microbiota (HFM) from stunted toddler (stunted Gn Pig, *n* = 8); and (4) Gn piglets transplanted with HFM from normal toddler (normal Gn Pig, *n* = 11). To assess the microbial community composition post transplantation, each group of HFM Gn piglets were further subdivided into two, where a subset of pigs was euthanized at PTD7 and the rest at PTD23. Notably, one of the toddlers (6_CP04824) grouped as stunted (clinically) had gut microbiota composition, diversity and functions similar to healthy toddlers (Fig. 2 and S2), hence we excluded the data from further comparative analysis.

### Microbiota diversity among toddlers and HFM Gn pigs

Microbiota diversity is the measure of how many different species exist and how evenly distributed they are within the community. In the gut, lower diversity is considered a marker of dysbiosis (microbial imbalance).

**Fig. 1:**
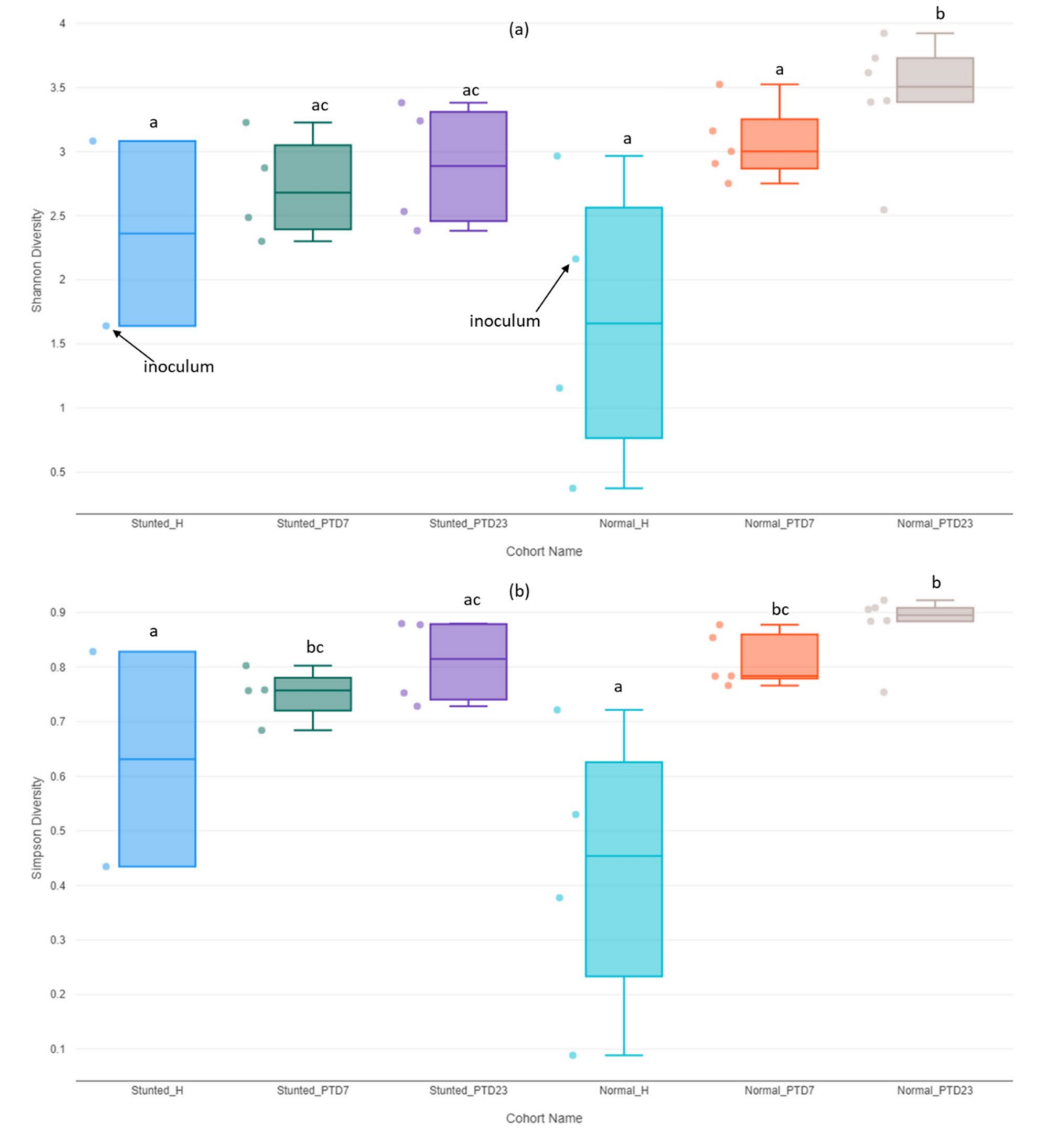
Box and whiskers plot for estimated alpha diversity indices (a– Shannon and b– Simpson) in the feces of toddlers (normal/stunted) and large intestinal content bacterial species of HFM Gn piglets at PTD7 and 23. Statistical comparisons were performed using the Wilcoxon rank-sum test. Boxes with different letters indicate statistically significant differences (*p* < 0.05)

**Alpha diversity indices** We observed no significant difference in α-diversity indices (Simpson, considering both the number of species and their evenness, and Shannon, accounting for the number of species present, as well as the relative abundance of each species) between the healthy and stunted toddler groups (Fig. S1a). However, HFM transplantation resulted in a significantly higher (*p* < 0.05) diversity of bacterial species in the large intestinal contents of Gn piglets transplanted with normal HFM as compared to those transplanted with stunted HFM on PTD23 (Fig. 1). Thus, although not detectable in toddlers, HFM transplantation of Gn piglets revealed higher or significantly higher diversity of microbial composition in normal vs. stunted HFM samples.

**Fig. 2:**
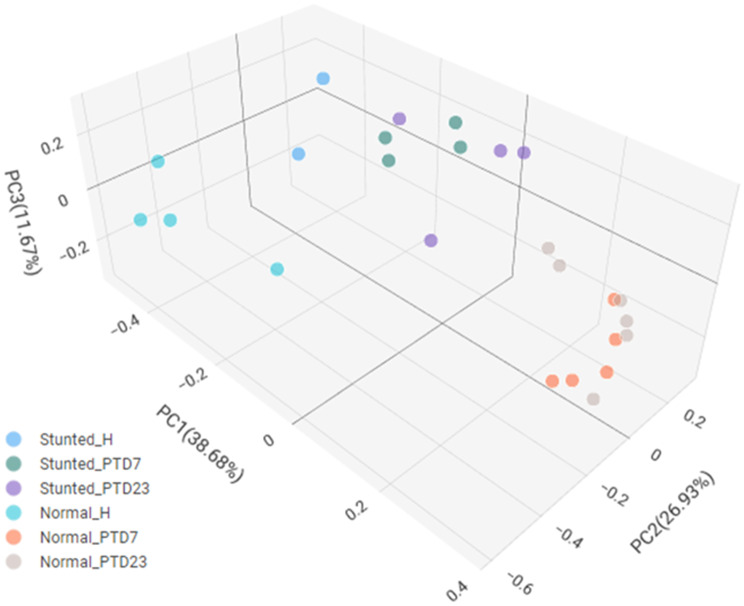
Principal coordinate analysis (PCOA) showing beta diversity in fecal bacteria species from normal and stunted children and large intestinal content of HFM Gn piglets. Normal_H = normal toddlers; Stunted_H = stunted toddlers; Stunted_PTD7/23 = Gn piglets transplanted with fecal material from stunted toddler fed at day 7 and 23; Normal_PTD7/23 = Gn piglets transplanted with fecal material from normal toddler at day 7 and 23

**Beta diversity index** is a measure of the similarity or dissimilarity of species between communities. Bray-Curtis dissimilarity considers both size (overall abundance per sample) and shape (abundance of each taxon) of the communities. This index reflects the differences in bacterial community composition and structure across different samples. There was no significant difference in beta diversity index between bacteria species in normal and stunted toddlers (Fig. S1b), while Gn pig large intestinal contents and toddler fecal bacterial composition differed significantly (Fig. 2).

### Microbial composition in toddlers and the HFM Gn pigs

**Fig. 3:**
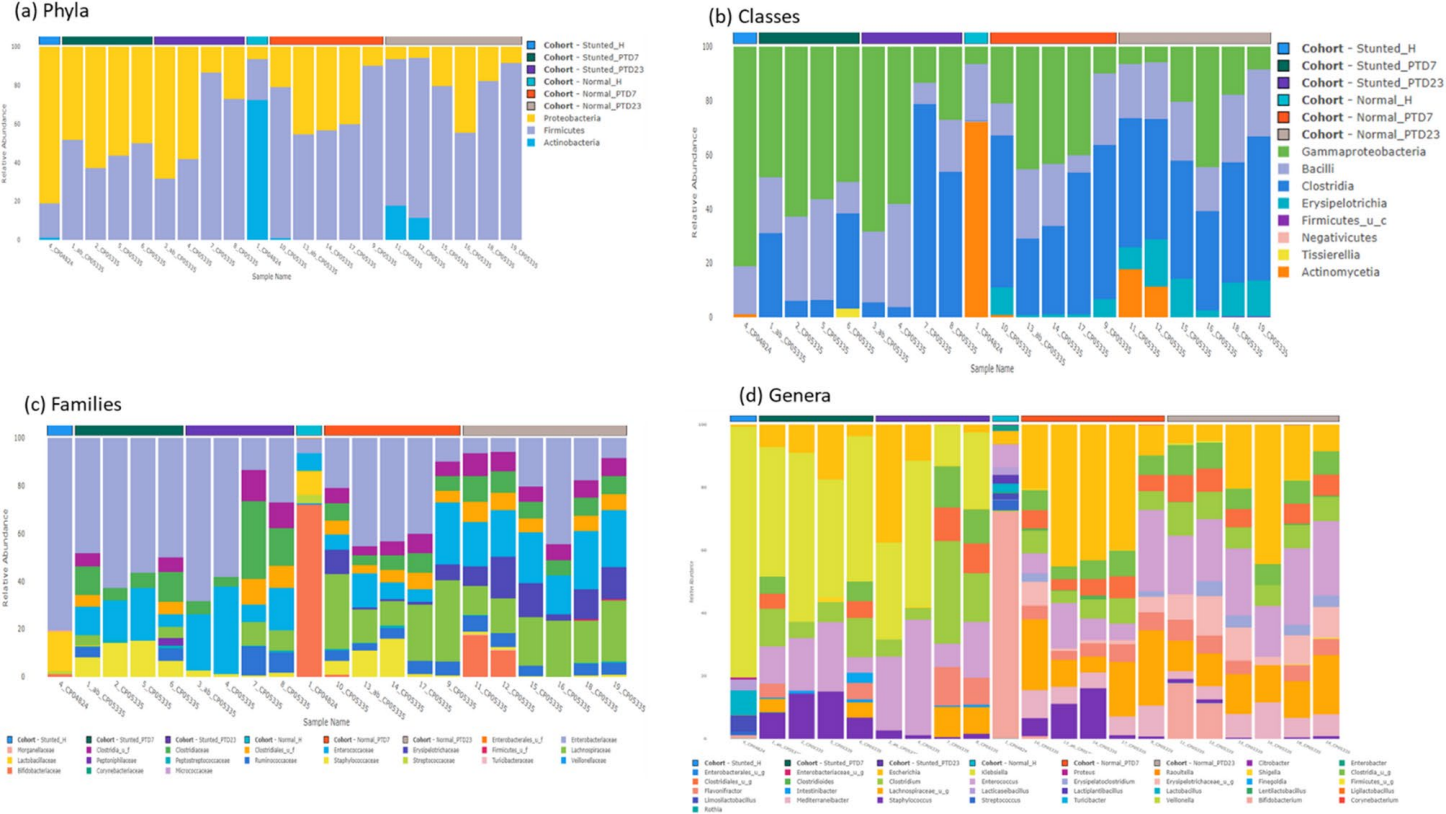
Stacked bar plots of relative abundance of the phyla (**a**), class (**b**), families (**c**) and genera (**d**) of bacteria in feces of normal and stunted toddlers, and large intestinal content of HFM Gn piglets

Although there were no significant differences in bacterial diversity indices between healthy/normal vs. malnourished/stunted toddlers, there was a marked difference in bacterial composition. Feces from normal toddlers had predominately bacterial species from the *Actinobacteria* phylum (53–98%) followed by *Firmicutes* (21–37%) and *Proteobacteria* (2–9%), while feces from stunted toddlers had predominantly *Proteobacteria* (44–81%) followed by *Firmicutes* (18–21%) and *Actinobacteria* (1–34%) (Figure S2a). Similarly, the large intestinal contents of Gn piglets transplanted with fecal material from a stunted child had *Proteobacteria* and *Firmicutes* predominating at PTD7, with *Firmicutes* predominating over *Proteobacteria* at PTD23. The large intestinal content of Gn piglets transplanted with feces from a normal child had predominately *Firmicutes* throughout the experiment (Fig. 3a). Thus, our HFM experimental transplantation of Gn piglets demonstrated that with the exception of the *Actinobacteria* phylum, the major differences at the phylum level were transferable and consistent.

In terms of bacterial classes (Fig. 3b), *Actinomycetia* was dominant in the feces of normal toddlers followed by *Bacilli*, while the feces of stunted toddlers had predominately *Gammaproteobacteria* followed by *Bacilli.* Similarly, *Gammaproteobacteria*,* Clostridia* and *Bacilli*, were the most abundance classes in the large intestinal contents of HFM stunted-Gn piglets. On the other hand, *Clostridia* predominated the large intestinal contents of HFM normal-piglets, followed by *Gammaproteobacteria* and *Bacilli*, respectively. *Erysipelotrichia* and *Actinomycetia* were present in the large intestinal contents of HFM normal-Gn pigs. Although *Actinomycetia* were abundant in the feces of the normal toddler, their growth was reduced in the Gn piglets. Additionally, *Clostridia* was undetectable in both normal and stunted toddlers; however, their abundance was increased in the HFM transplanted Gn pigs, especially in the HFM normal-Gn pigs.

Further, the *Bifidobactericeae* family (53–98%) was the most abundant in the feces of normal toddlers followed by *Enterococcaceae* (7–19%) and *Lactobacillaceae* (3–10%), while in the stunted toddlers, bacteria of the *Enterobacteriaceae* family (44–80%) were predominate followed by *Bifidobactericeae*,* streptococcaceae*,* Lactobacillaceae* (Fig. S2b). Similar to the stunted toddler fecal samples, bacterial species of the *Enterobacteriaceae* family (42–54%) were the most abundant in the large intestinal contents of HFM stunted-Gn piglets, followed by *Enterococcaceae* (14–21%), *Clostridiaceae* family (9-14.4%), *Staphylococcaceae* (1.5–11%), *Lachnospiraceae* (2–5%), and *Ruminococcaceae* (2–5%), with *Bifidobactericeae* undetectable. The large intestinal contents of HFM normal-Gn piglets had predominantly bacteria species of *Enterobacteriaceae* (17–32%), followed by *Lachnospiraceae* (19-22.5%), *Enterococcaceae* (12-20.7%), *Erysipelotrichaceae* (4-11.4%), *Staphylococcaceae* (7%), *Clostridiaceae* family (5–8%), and *Ruminococcaceae* (4.5%) families, with *Bifidobactericeae* family comprising only 4.8% (Fig. 3c).

At the genus level, *Bifidobacterium* (53–98%) and *Enterococcus* (7–19%) predominated in the feces of healthy/normal toddlers, while in the stunted toddlers, the *Klebsiella* (32–80%), *Bifidobacterium (1–34%)*,* streptococcus (1–18%)* and *Lactobacillus* (0.5-8%) genera were the most abundant (Fig. S2c). The normal toddlers gut microbiota was predominantly *Bifidobacterium longum* while *Klebsiella pneumoniae* was dominant in stunted toddlers (Fig. S2d). The bacterial genera in Gn piglets were more diverse with *Klebsiella* (29–45%) and *Escherichia* (17–32%) genera dominating in stunted-Gn pigs, (Fig. 3d). This was followed by *Clostridium* (15–25%; 17–22%), *Enterococcus* (14–21%; 12–21%) and *Staphylococcus* (1.5–11%); 0.6-7%). Although *Bifidobacterium* was abundant in normal toddler feces, its growth was reduced in the large intestine of HFM transplanted Gn pigs. Similarly, the growth of *Lactobacillus* and *Bifidobacterium* that was present in the feces of the stunted toddler was reduced in Gn pigs. Overall, the bacterial composition of the stunted toddlers’ fecal microbiota was more reflected in large intestinal content of Gn pigs compared to that of the normal toddler.

### Microbial community composition of human fecal microbiota post-transplantation in the Gn pig model

**Fig. 4:**
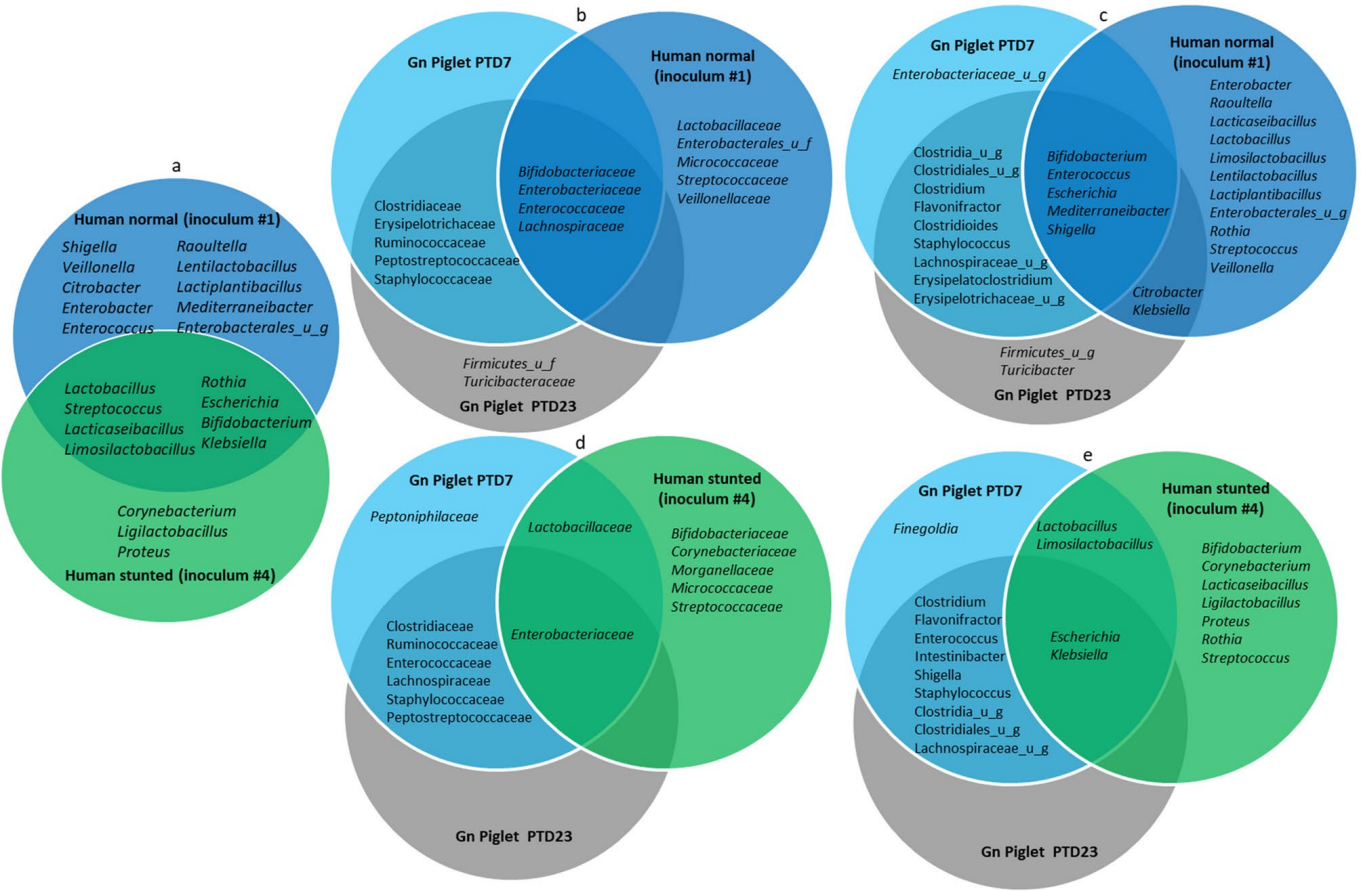
Venn diagrams showing unique/shared families (**b**, **d**) and genera (**a**, **c**, **e**) of bacteria present in the normal (blue) and stunted (green) toddlers inocula and large intestinal contents of gnotobiotic piglets (light blue and grey) transplanted with feces from normal (**b**, **c**) and stunted (**d**, **e**) toddlers

Group-specific core genera among toddlers’ fecal microbiota revealed 8 unique genera (*Corynebacterium*,* Ligilactobacillus*,* Proteus*) in the malnourished/stunted toddlers, 19 in the healthy/normal toddlers, and 8 that overlapped between both groups (Fig. S3a). The genera shared between normal toddler and the HFM Gn pigs, but not between stunted toddler and the fecal HFM Gn pigs were *Bifidobacterium*, *Enterococcus*,* Mediterraneibacter*,* Shigella* and *citrobacter* (Fig. 4c, e). Only *Lactobacillus* and *Limosilactobacillus* colonized Gn pigs from the stunted toddler, but not from the normal toddler (Fig. 4e).

**Fig. 5:**
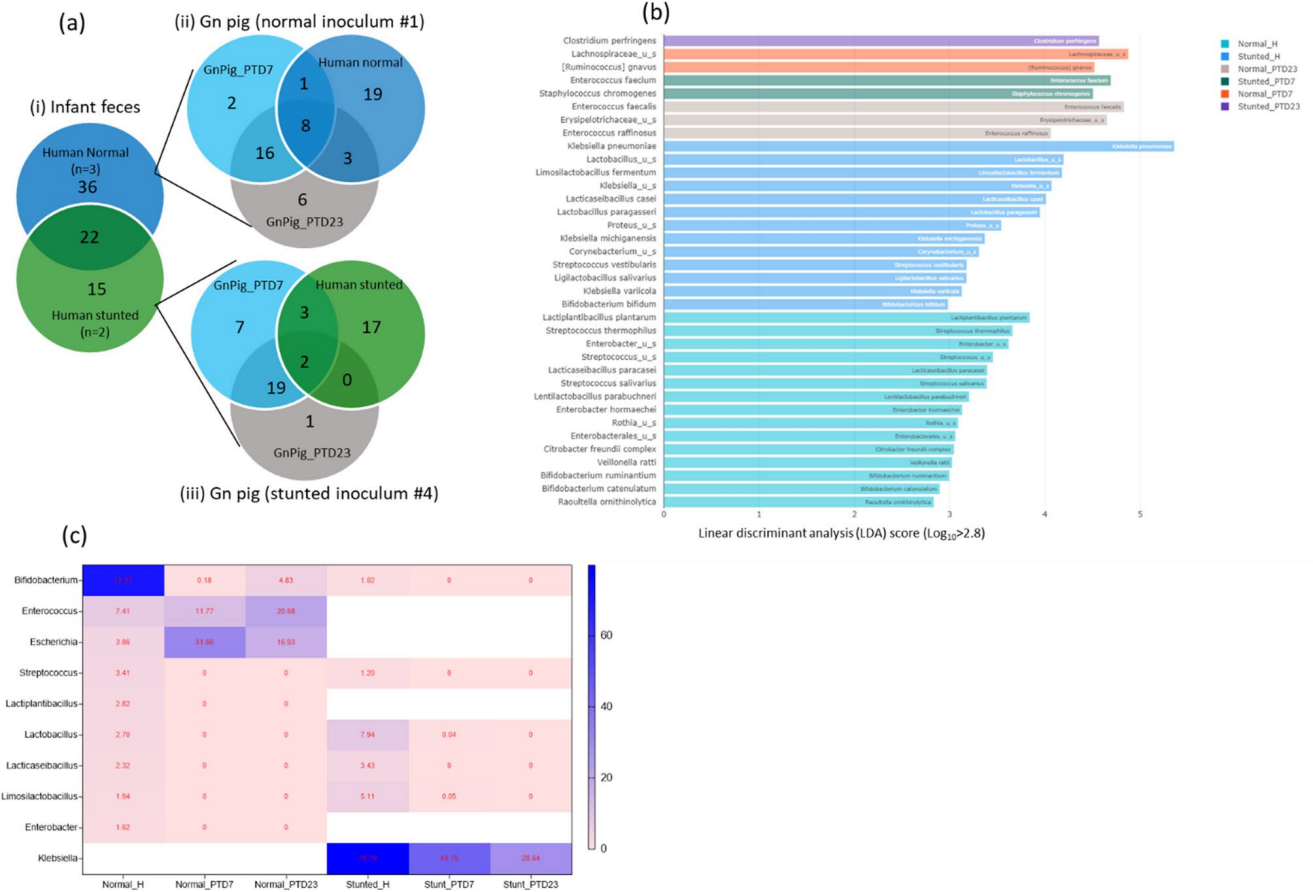
(**a**) Venn diagram based on number of bacterial species unique/shared among toddlers and HFM Gn piglets. (**b**) Histogram illustrating significantly different enriched bacterial species among the cohorts in LDA Effective Size (LEfSe) analysis (*p* < 0.05). (**c**) Heatmap showing top bacterial genera (> 1% relative abundance) in inoculum (normal and stunted) and success of their colonization in Gn pigs at PTD7 and PTD23, white color indicate that the bacteria genera were absent in the toddler’s inoculum

A large number of bacterial species from normal toddler feces (*n* = 36) and stunted toddler feces (*n* = 32) colonized the large intestinal contents of HFM Gn pigs (Fig. 5a). However, we also observed several bacterial species in large intestinal contents of Gn piglets transplanted with feces from normal (*n* = 24) and stunted (*n* = 27) toddlers that were not detectable in the original fecal samples (Fig. 5a). Comparison of the core OTUs by LDA effect size (LEfSe) identified 3 (*B. longum*, *S. dysenteriae* and *S. thermophilus*) and 7 species to be significantly discriminative, with an absolute LDA score > 2.5, between the normal and stunted toddlers, respectively (Fig. S3b). Moreover, comparison of the core OTUs by LEfSe (LDA score > 2.8) identified 15, 13 and 8 features to be significantly discriminative between the normal toddler, stunted toddler and HFM inoculated Gn piglets, respectively (Fig. 5b). *Streptococcus thermophilus* and *Lactiplantibacillus* (LDA > 3.6) were highly enriched in the normal toddler feces, while *K. pneumoniae* and *Lactobacillus spp* (LDA > 4.2) were highly enriched in the stunted toddler. Of the 8 species enriched in Gn piglets (LDA > 4.0), *Lachnospiraceae_u_s* and *Enterococcus faecalis* (LDA > 4.8) were highly enriched followed by *Enterococcus faecium* (LDA 4.7) and *C. perfringens* (LDA 4.6) (Fig. 5b). Of note, most enriched species both in toddlers and Gn pigs are bacteria belonging to pathogenic species. Similarly, we observed that most of the beneficial commensals abundant in the normal toddler (e.g. *Bifidobacterium*) did not establish well in the Gn pigs compared to the pathogenic bacteria such as *Enterococcus* and *Escherichia* (Fig. 5c). A similar pattern of bacterial species was observed in the stunted cohorts (Fig. 5c).

### Bacterial virulence factors and antimicrobial resistance genes identified in toddlers and HFM Gn pigs

**Fig. 6:**
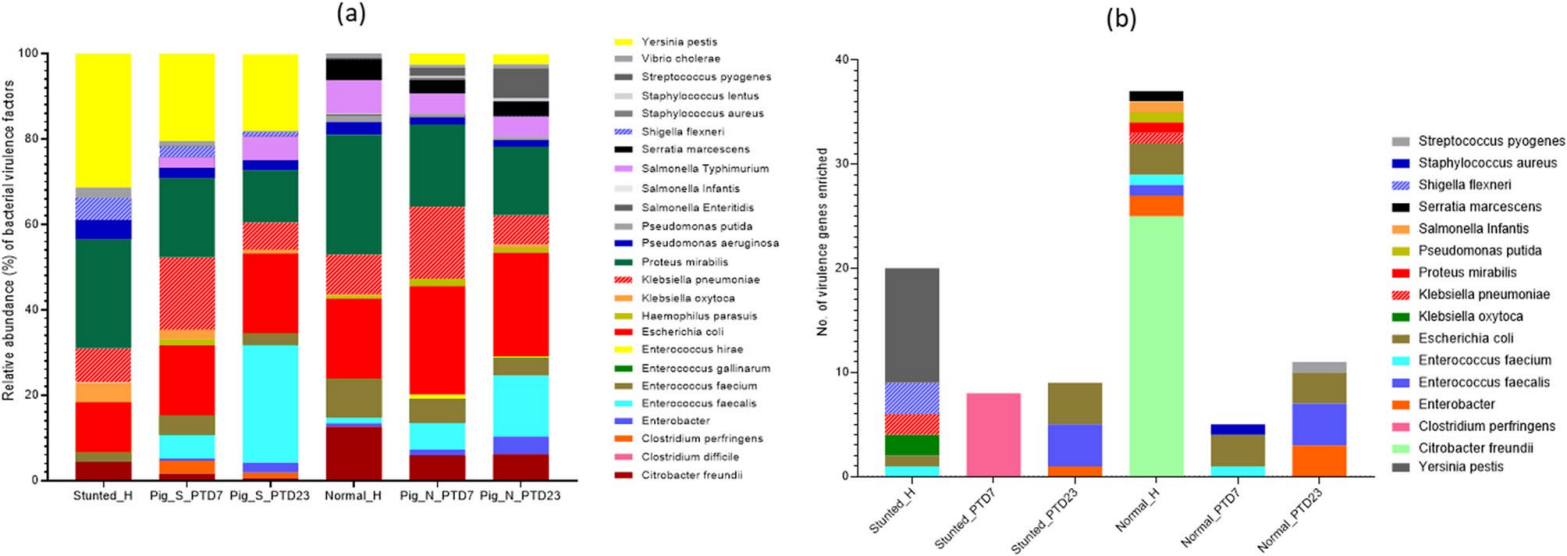
Stacked bar plots illustrating relative abundance of bacterial virulence factors among the cohorts (**a**) and number of bacterial virulence genes (**b**) enriched among the cohorts in LDA Effective Size (LEfSe) analysis (*p* < 0.05)

**Virulence factors** - Virulence factors in pathogens are involved in the various stages of infection, including the production of toxins, colonization, adhesion and invasion, as well as survival inside the host cells. Gut bacterial virulence factors have complex effects on gut infections. The precise mechanisms of these interactions are poorly understood; however, bacterial virulence factors may impact GI infections through competition for resources, modulation of the immune response, alteration of gut barrier function, and production of toxins. We used VFDB (Virulence Factor Database) to analyze virulence factors in microbiome of toddlers and Gn pigs without targeting specific pathogens but aimed to broadly compare virulence potential between groups. The composition of bacterial virulence factors observed in feces of normal and stunted toddlers were similar (except for *Y. pestis* and *S. flexineri* virulence factors which were present only in stunted toddlers) (Fig. 6a and Fig. S4a). However, the relative abundance of these factors was markedly different between the two toddler groups, with *P. mirabilis* and *E. coli* virulence factors predominating in normal toddler feces while *Y. pestis* and *P. mirabilis* virulence factors dominating stunted toddler feces (Fig. S4a). Analysis of enriched virulence genes in normal and stunted toddlers showed that the majority of enriched genes in both cohorts were from pathogenic bacteria (Fig. S4b). In HFM Gn pigs, we observed a similar pattern as for the original inoculum, suggesting that virulence factors were readily transferable from human fecal microbiota to Gn pigs. Analysis of enriched virulence genes (based on LDA > 2.5) revealed that normal/healthy toddler [10] had more enriched virulence genes compared to stunted toddler [6] and HFM Gn piglets [5] (Fig. 6b; Table 1). The top 5 virulence genes highly enriched in feces of normal toddler (LDA > 3.5) included *K. pneumoniae* tnpA, *Serratia marcescens* orfA, *Escherichia coli stbA* and *C. freundii* orf39/41, whereas in stunted toddler feces, only *Y. pestis* genes (irp1, irp2 ybtU, fyuA & *ybtE*) were in the top 5 highly enriched genes (LDA > 4.0). *E. faecalis and E. coli* genes were the only highly enriched bacterial virulence genes in large intestinal contents of HFM stunted-Gn piglets at PTD23, while only *C. perfringens* gene*s* were enriched (LDA > 4.0) at PTD7. *E. coli (AAC6-Ib*,* tnpR*,* OXA-1)*,* E. faecium* (ermB) and *S. aureus* (33390919) genes were highly enriched (LDA > 3.0) in HFM normal-Gn pigs at PTD7, while *S. pyogenes (*52345264), *E. coli* (stbA, traA) and *E. faecalis* (32470452, 32470456) genes were the top 5 highly enriched virulence genes at PTD23 in this cohort (Fig. 6b; Table 1).

**Fig. 7:**
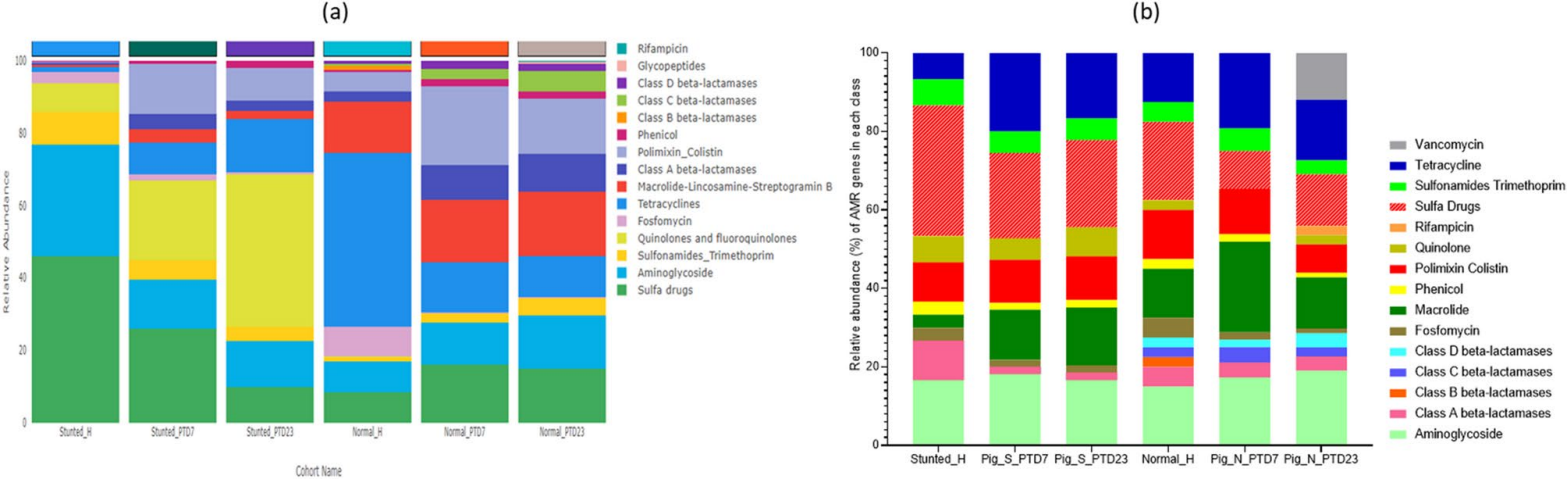
Stacked bars showing relative abundance of AMR bacterial classes (**a**) and genes (**b**) among the toddlers and HFM Gn pigs cohorts

Of interest, there were fewer bacteria virulence factors enriched in large intestinal contents of Gn pigs transplanted with feces from normal/stunted toddlers compared to the virulence factors detected in the original inoculum. Furthermore, the virulence genes enriched in the HFM Gn pig groups were mostly from pathogenic bacteria (Table 1). Only *E. faecalis* and *C. perfringens* virulence genes were the topmost enriched in large intestinal contents of HFM stunted-Gn pigs. These bacteria have been shown to carry resistance genes to many antibiotics, as also observed in AMR analysis (Fig. 7).

**Table 1:** Summary of number of bacterial virulence and AMR genes enriched among the cohorts in LDA effective size (LEfSe) analysis (*p* < 0.05)

Groups	Species	No. of virulence genes	Antibiotic classes	No. of AMR genes
Normal/healthy toddler	*Citrobacter freundii*	25	Aminoglycoside	1
	*Enterobacter*	2	Class B beta-lactamases	1
	*Enterococcus faecalis*	1	Fosfomycin	2
	*Enterococcus faecium*	1	Macrolide	3
	*Escherichia coli*	3	Quinolone	1
	*Klebsiella pneumoniae*	1		
	*Proteus mirabilis*	1		
	*Pseudomonas putida*	1		
	*Salmonella Infantis*	1		
	*Serratia marcescens*	1		
		**37**		**8**
Normal Gn pigs (PTD7)	*Enterococcus faecium*	1	Aminoglycoside	1
	*Escherichia coli*	3	Macrolide	1
	*Staphylococcus aureus*	1	Phenicol	1
			Polimixin Colistin	1
			Sulfa Drugs	2
Normal Gn pigs (PTD23)	*Enterobacter*	3	Aminoglycoside	2
	*Enterococcus faecalis*	4	BACTRIM	2
	*Escherichia coli*	3	Class A beta-lactamases	1
	*Streptococcus pyogenes*	1	Macrolide	2
			Class C beta-lactamases	1
Stunted toddler	*Enterococcus faecium*	1	Aminoglycoside	2
	*Escherichia coli*	1	BACTRIM	1
	*Klebsiella oxytoca*	2	Class A beta-lactamases	2
	*Klebsiella pneumoniae*	2	Fosfomycin	1
	*Shigella flexneri*	3	Quinolone	2
	*Yersinia pestis*	11	Sulfa Drugs	7
		**20**		**15**
Stunted Gn pigs (PTD7)	*Clostridium perfringens*	8	Tetracycline	1
Stunted Gn pigs (PTD23)	*Enterobacter*	1	Aminoglycoside	1
	*Enterococcus faecalis*	4	Macrolide	1
	*Escherichia coli*	4	Phenicol	1

**Antimicrobial resistance (AMR)–** Our data demonstrated that stunted children had higher numbers of bacteria carrying antibiotic resistance genes (*N* = 15) compared to normal children (*N* = 8) (Table 1), suggesting that stunted children may have been treated with antibiotics repeatedly or for longer periods of time. Of interest, these AMR resistant bacteria in the stunted toddler were poorly transferrable to Gn piglets, compared to those identified in the feces of the normal toddler.

The composition of AMR classes and genes were similar between normal and stunted toddlers; however, we observed marked difference in their relative abundance between these groups (Fig. S5a). The feces of the normal toddler had predominately bacteria resistant to antibiotics that belonged to Tetracycline and Macrolide-Lincosamine-Streptogramin B classes, while bacteria resistant to antibiotics in Sulfa and Aminoglycoside classes were most abundant in feces of the stunted toddler (Fig. S5a). These major classes were established in HFM Gn piglets; however, their abundance and proportions varied. Large intestinal contents of HFM stunted-Gn piglets were enriched for bacteria resistant to antibiotics in Quinolones and fluoroquinolone (22–42%), Sulfa (10–26%) and Aminoglycoside (12.6–13.6%) classes (Fig. 7a). Whereas HFM normal-Gn pigs had predominately bacteria resistant to Polimixin (15.4–21.6%), Macrolide-Lincosamine-Streptogramin B (17%) and Sulfa drugs (15–16%). Of note, bacteria resistant to Rifampicin, and Glycopeptides were only present in HFM normal-Gn piglets, although these AMR classes were not detectable in the original inoculum.

Bacteria carrying genes resistant to Aminoglycoside and Sulfa drugs predominated in the feces of the stunted toddler and in HFM stunted-Gn piglets (Fig. 7b; Table 1). Bacteria resistant to Tetracycline, Macrolides, Class A beta-lactamases and Polimixin_Colistin were also enriched in the stunted child feces and were transferrable to Gn piglets. In comparison the normal toddler feces had predominately bacteria resistant to Sulfa drugs followed by Aminoglycoside, Tetracycline, Polimixin_Colistin and Macrolide resistant bacteria. Although the bacteria carrying Tetracycline and Polimixin_Colistin resistance genes were lower in the stunted toddler, the large intestinal contents of the HFM stunted-Gn piglets had a high abundance of bacteria carrying genes resistant to these antibiotics (Fig. 7b; Table 1). Comparison of the bacterial AMR genes by linear discriminant analysis effect size (LEfSe) identified 8, 15, 14, and 4 AMR genes to be significantly discriminative, with an absolute LDA score > 3.5, between the normal toddler, stunted toddler, HFM normal-Gn piglets, and HFM stunted-Gn piglets, respectively (Fig. S6). These contrasting findings suggest that various host factors are likely to contribute to microbiome composition and diversity.

### Functional features of bacteria present in toddler feces and large intestinal contents of HFM Gn pigs

**Fig. 8:**
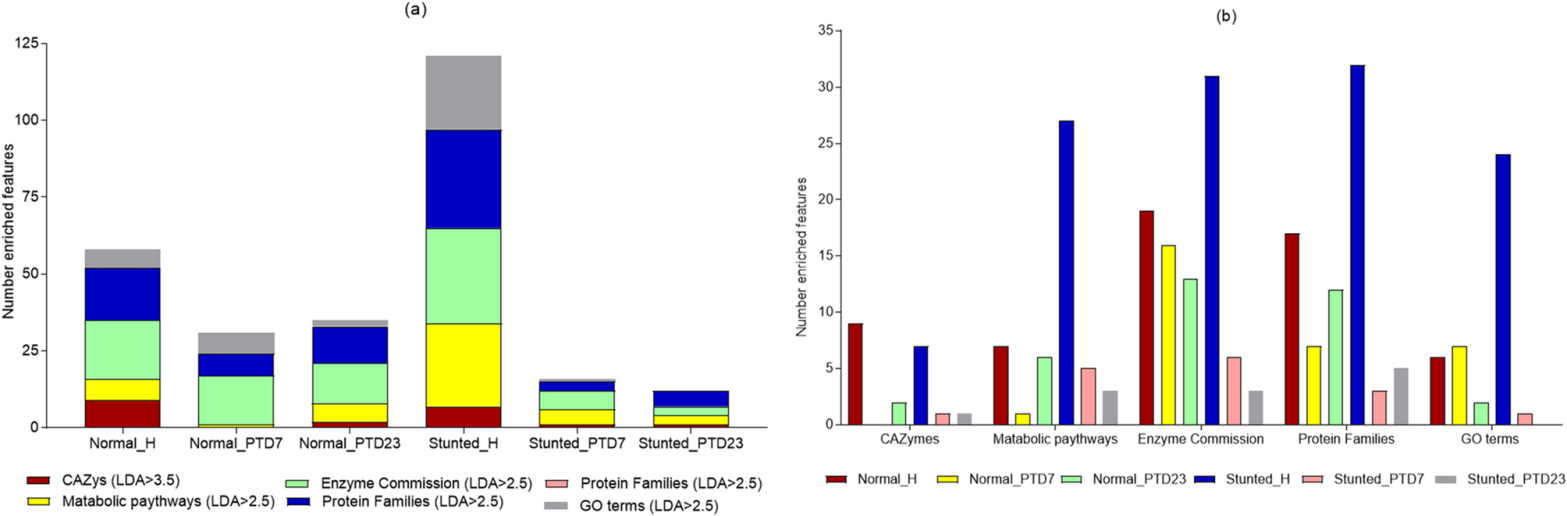
Summary of the number of enriched bacterial functional features within the cohorts (**a**) and among the cohorts (**b**)

Here we present a summary of functional similarities and differences between the gut microbiota present in the feces of normal and stunted toddlers and in the large intestine of the toddler microbiota transplanted Gn piglets (Fig. 8a and Fig. S7a). Gut microbiota functions analysed were carbohydrate active enzymes (CAZymes), metabolic pathways, protein families (pfam), enzyme commissions (EC) and gene Ontology (GO) terms. In general, functional analysis revealed that enzymes involved in carbohydrate biosynthesis and metabolic pathways related to protein/amino acid, carbohydrate and fat catabolism were highly enriched in the stunted toddler feces (Fig. S7), whereas enzymes involved in carbohydrate degradation and anabolic pathways were enriched in normal toddler feces (Figs. 8 and 10). Generally, similar trends were observed in the HFM Gn pigs. As a result of the reduced abundance of many beneficial members of the known bacterial phylotypes in the malnourished/stunted compared to the healthy toddlers, we speculated that there was an enrichment of most of the carbohydrate biosynthesis bacterial functions to be able to utilize the few nutrients available in malnourished/stunted toddlers’ diet (Fig. 8b). Overall, we observed that in healthy toddlers, gut bacteria degrade dietary carbohydrates to provide host energy and support bacterial growth (anabolism). While in stunted toddlers, gut bacteria scavenge limited resources (catabolism) and synthesize structural carbohydrates (biosynthesis) to survive, disrupting host energy balance.

**Fig. 9:**
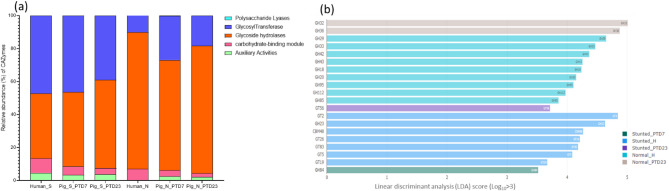
(**a**) Stacked bars showing relative abundance of carbohydrate active enzymes (CAZys) among the cohorts. (**b**) Histogram illustrating significantly different enriched CAZys among the cohorts in LDA Effective Size (LEfSe) analysis (LDA > 3.5; *p* < 0.05)

**Carbohydrate active enzymes (CAZymes)** are critical in building and breakdown of complex carbohydrates and glycoconjugates such as cellulose, hemicellulose, and starch. Based on amino acid sequence similarities, these enzymes are categorized as glycoside hydrolases (GHs), glycosyltransferases (GTs), polysaccharide lyases (PLs), carbohydrate esterases (CEs) and the non-enzymatic species carbohydrate-binding modules (CBMs) and Auxiliary Activities (AAs). Carbohydrate active enzymes (CAZymes) analysis revealed that microbiota enzymes involved in carbohydrate biosynthesis (GTs) were abundant in stunted toddlers feces (Fig. S7a and Fig. 9a), indicating limited energy availability and substrate diversity in the gut; whereas those involved in carbohydrate degradation (GHs) were abundant in healthy/normal toddlers feces, reflecting higher diversity and capacity for metabolizing complex carbohydrates in a nutrient-rich environment (Fig. S7a and Fig. 9a). A similar trend was observed in HFM Gn pigs (Fig. 9a). Comparable results were obtained when we carried out LEfSe analysis; whereas GT2 was highly enriched in the stunted toddlers, GH29 was enriched in the healthy toddlers (Fig. 9b).

**Fig. 10:**
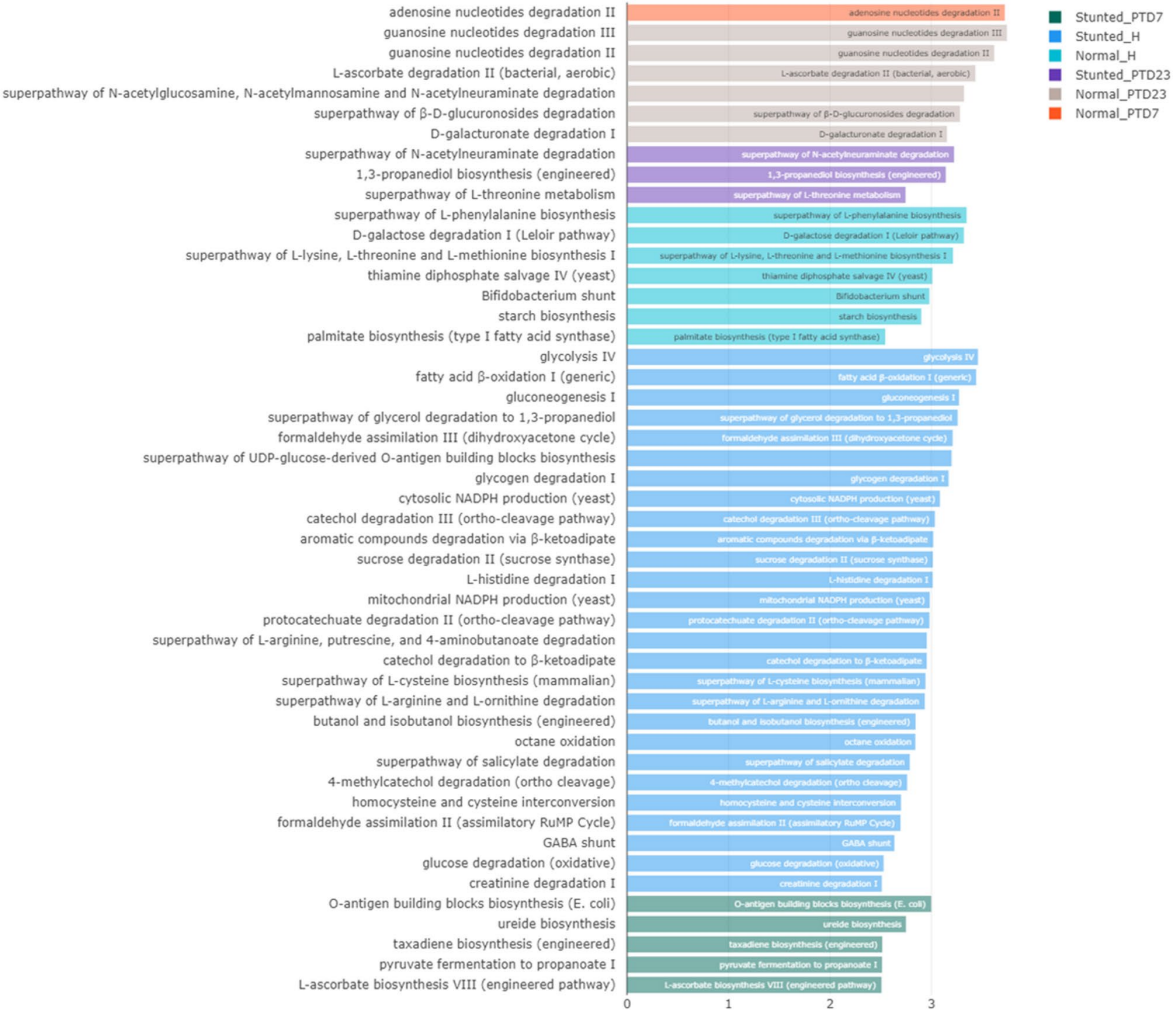
Histogram illustrating significantly different enriched metabolic pathways among the cohorts in LDA Effective Size (LEfSe) analysis (LDA > 2.5; *p* < 0.05)

**Bacterial metabolic pathways** consist of a series of enzymatic reactions that cause the alteration of a substrate multiple times before arriving at the final product. This includes catabolism (breaking down complex macromolecules to their basic components to obtain energy), and anabolism (formation of new products with the use of simple molecules and energy). Contrary to CAZymes results, we observed that, in the malnourished/stunted toddlers’ feces, metabolic pathways that were highly enriched involved catabolism, whereas pathways involved in anabolism were enriched in the healthy/normal toddler feces (Fig. S7). However, these trends were reversed in HFM Gn pigs where biosynthesis pathways were enriched in large intestinal contents of Gn pigs transplanted with stunted toddler feces, while degradation metabolic pathways were enriched in Gn pigs transplanted with healthy/normal toddler feces (Fig. 10 and Fig. S8).

**Enzyme commissions (EC), protein family (pfam) and gene ontology (GO)**: Comparable trends were observed in the analysis of EC, pfam and GO data for toddlers’ cohort and the HFM Gn pig groups (Fig. S9– SS14). Analysis of ECs revealed higher prevalence of enzymes linked to glycolysis, the TCA cycle, and oxidative phosphorylation in healthy toddlers, reflecting efficient energy metabolism. However, in stunted toddlers, enzymes favoring anaerobic fermentation processes dominated, indicative of impaired microbial nutrient provision to the host (Fig. S9-S10). In healthy toddlers, there was abundance of protein families associated with carbohydrate and lipid metabolism, nutrient transport, and vitamin biosynthesis, whereas in stunted toddlers there was Increase in stress-response proteins and those linked to survival under nutrient-limiting conditions (Fig. S11-S12). Enriched GO terms for metabolic process, biosynthetic process, and cellular component organization were observed in healthy toddlers’ microbiota, suggesting a robust microbial community contributing to homeostasis and nutrient cycling. While in stunted toddlers, dominant GO terms included response to stress and pathogenesis, highlighting a shift toward microbial survival and host interaction under adverse conditions (Fig. S13-S14).

### HFM influence on blood routine indices

To exemplify how distinct HFM composition and functions impact the host physiology, we compared blood cell counts of the HFM transplanted piglets. The blood routine indices of the HFM transplanted Gn pigs showed variations in red blood cells (RBC), white blood cells (WBC), lymphocytes, monocytes, neutrophils, hemoglobin, and platelets across different time points and between normal and stunted groups. In general, the counts of most white blood cells (including total WBCs, lymphocytes, monocytes, neutrophils and platelets) were increased in pigs transplanted with normal microbiota (Table 2). However, counterintuitively, RBC counts, and hemoglobin levels were consistently higher in piglets with stunted microbiomes, suggesting that the malnourished microbiome may induce physiological changes that lead to increased red blood cell production. Notably, there were relatively higher WBC and neutrophil counts in HFM normal-Gn pigs compared to HFM stunted-Gn pigs at PTD 13, suggesting that the healthy microbiome may induce a more robust immune function, even in the steady state. These hematological changes reflect the systemic effects of gut microbiota on host physiology, potentially influencing immune responses and overall health [42]. Besides hematological changes observed, we compared the piglets weights, but no significant differences were observed (Table 2). Similar findings in human studies have shown correlations between gut microbiota composition and immune cell profiles [43, 44]. By elucidating the mechanisms behind these changes, we may be able to develop novel therapeutic strategies to address malnutrition and related disorders. Hence, more research is necessary to fully understand the complex interactions between the gut microbiome and the immune system.

**Table 2:** Effects of gut microbiota on the blood routine indices in a humanized Gn pig model

	PTD7		PTD13		PTD23	
Parameters	Stunted	Normal	Stunted	Normal	Stunted	Normal
RBC (10^9/L)	5.64 ± 0.33	4.72 ± 0.72	6.69 ± 0.16	6.48 ± 0.14	6.14 ± 0.11	5.96 ± 0.36
WBC (10^9/L)	6.15 ± 0.84	9.57 ± 2.33	**6.97 ± 0.71** ^**a**^	**13.78 ± 2.91** ^**b**^	6.38 ± 0.36	7.84 ± 1.74
Lymphocytes (10^9/L)	4.34 ± 0.63	4.34 ± 0.82	4.79 ± 0.41	4.99 ± 0.51	2.99 ± 0.18	3.10 ± 0.35
Monocytes (10^9/L)	0.06 ± 0.01	0.08 ± 0.02	0.06 ± 0.02	0.12 ± 0.03	0.07 ± 0.01	0.09 ± 0.02
Neutrophils (10^9/L)	1.76 ± 0.25	5.16 ± 1.75	**2.11 ± 0.4** ^**a**^	**8.67 ± 2.75** ^**b**^	3.32 ± 0.21	4.65 ± 1.39
Hemoglobin (g/dL)	8.21 ± 0.41	7.38 ± 0.75	9.10 ± 0.17	8.38 ± 0.43	7.36 ± 0.37	6.75 ± 0.55
Platelet (10^9/L)	574.86 ± 52.96	366.90 ± 94.21	361.50 ± 81.95	455.00 ± 65.81	**458.30 ± 31.96** ^**a**^	**583.83 ± 77.01** ^**b**^
Weight (lb)	4.13 ± 0.23	3.95 ± 0.13	5.75 ± 0.25	5.08 ± 0.20	7.63 ± 0.24	7.42 ± 0.37

Stunted– Gn pigs inoculated with fecal gut microbiota from a stunted toddler; normal - Gn pigs inoculated with fecal gut microbiota from a normal/clinically healthy toddler; PTD– post transplantation day; RBC– red blood cells; WBC– white blood cells. Values are presented as mean ± SE. **Bold** values highlight the parameters that showed significant differences (*p* < 0.05) between Gn pigs transplanted with normal vs. stunt fecal material

## Discussion

The gut microbiota plays a critical role in maintaining host health by facilitating nutrient metabolism, modulating the immune system, and providing protection against pathogens [8, 11]. However, disruptions in this microbial balance, known as dysbiosis, can increase susceptibility to infections and impact host-microbe interactions [20, 21, 45]. We aimed to characterize the composition, diversity and functional characteristics of fecal microbiota of malnourished (stunted) versus clinically healthy/normal toddlers (donors) and to explore the transferability of structure of these bacterial communities using a gnotobiotic (Gn) pig model. The use of Gn pigs as a model for studying human gut microbial community profile is innovative. While prior research may have focused on static snapshots of microbiota composition, this study’s longitudinal design offers insights into how these microbial communities establish and evolve over time in a controlled environment (germ-free condition). Besides, in addition to previously investigated compositional differences, our current study assessed the functional implications of these differences between healthy and malnourished/stunted toddlers. Likewise, we have confirmed that our model recapitulates these ensuing functional characteristics, and they remain stable throughout the experiment. Thus, to the best of our knowledge this is the first attempt to link microbial community structure to physiological outcomes, providing a more holistic understanding of the microbiota’s role in health and disease. Another novel aspect of this study is that using toddlers’ microbiota [< 24months versus older children (24 + months and older) in other studies [46–48], we demonstrated that malnutrition-associated microbiological and physiological alterations initiate early in childhood, likely in infancy, before the microbiome matures reaching adult-like composition. Moreover, we identified specific bacterial taxa that are significantly enriched in healthy vs. stunted toddlers’ fecal microbiota and the transferability of these enriched taxa in the Gn pig model. This level of detail can pinpoint potential microbial biomarkers or therapeutic targets, which is a step forward from previous research that might have only noted general differences in diversity or broad taxonomic groups.

### Microbiota composition differs between clinically healthy vs. stunted toddlers

**Microbiota composition**: We identified distinct differences in the fecal bacterial composition between healthy and stunted toddlers. In the healthy toddler, there was a well-balanced diverse gut microbiota with beneficial bacteria, *Actinobacteria* predominating. These bacteria help in breaking down food, absorbing nutrients, and maintaining gut integrity. Conversely, stunted toddlers’ feces were dominated by pathogenic bacteria, *Proteobacteria*, with significantly lower proportions of beneficial bacteria, limiting the ability to properly digest and absorb nutrients. These findings align with previous research showing that malnutrition can alter gut microbiota composition, often increasing *Proteobacteria*, an indicator of dysbiosis, while decreasing beneficial bacteria like *Firmicutes* and *Actinobacteri*a [36, 49–51]. Previous studies have reported that malnutrition significantly alters gut microbiota, leading to a higher presence of potentially pathogenic bacteria and depletion of beneficial commensals, consistent with our study where stunted toddlers microbiota was dominated by *K. pneumonae* and deficient in *B. longum* [49, 52, 53]. Recently, Barrat et al. reported low abundance of *B. longum infantis* in Bangladeshi infants with severe acute malnutrition, and that administration of this bacteria to the affected infants improved their weight gain and reduced gut inflammation [54].

**Microbiota diversity**: We assessed the diversity of gut microbiota using alpha and beta diversity indices. Alpha diversity, which measures species variety and evenness within a community, showed significant differences between bacteria in toddler feces and large intestinal contents of HFM transplanted Gn piglets. The Shannon diversity index indicated notable differences in relative abundance across HFM Gn pig groups and not between toddler groups. Beta diversity, which compares species similarity between communities, also revealed significant differences among the gut bacterial communities of HFM Gn pigs and toddlers. Although our data did not show significant differences in diversity indices in gut bacteria between healthy and stunted toddlers, partly due to limited number of samples examined and age of the donors (< 2 yrs), previous studies using older children (3–5 yrs) have shown significant differences. Generally, higher microbial diversity correlates with a healthier gut ecosystem. The reduced alpha diversity observed in Gn pigs transplanted with HFM from the stunted toddler is consistent with other studies linking low microbial diversity to poor health outcomes, such as malnutrition and increased disease susceptibility [49, 52, 53]. A study comparing gut microbiota diversity between children from rural Africa and Europe, revealed that African children feeding on high fiber and low-fat diet, had higher gut microbiota diversity compared to children from Europe, consuming a Western diet high in fat and sugar [55]. This suggests that diet/nutritional status significantly influences gut microbiota diversity. Previous studies reported that Malawian infants with severe acute malnutrition (SAM) have significantly reduced alpha diversity, which correlates with impaired gut health and increased susceptibility to infections [56, 57]. Moreover, Yatsunenko et al. examining human gut microbial diversity in the USA, Venezuela and Malawi observed that microbial diversity increases with age and is influenced by diet, with lower diversity observed in malnourished populations [58]. Recently, Rothschild et al. also demonstrated that diverse diets and better nutritional status are associated with higher gut microbiota diversity, which supports the current study’s findings on the importance of microbial diversity for health [59]. Taken together, understanding the link between gut microbial diversity and nutritional status is crucial for developing effective interventions to improve child health.

### Microbial community profiles of healthy vs. stunted toddler fecal microbiota transplanted in Gn pigs

One of the challenges in studies using human microbiota-associated animal models is accurately representing the donor microbiota in the recipient animal. This study highlighted the gut microbial profiles of Gn pigs inoculated with fecal microbiota from either stunted or clinically healthy toddlers. Gn pigs receiving fecal microbiota from a stunted toddler exhibited an initial predominance of *Proteobacteria*, which later shifted to *Firmicutes* as they aged and consumed a nutritional replete diet. Conversely, those inoculated with fecal microbiota from healthy toddler consistently showed a predominance of *Firmicutes*. These findings mirrored the typical dominance of *Firmicutes* in healthy gut microbiota and the association of increased Proteobacteria with disease states [60]. Notably, the bacterial genera in Gn piglets were more diverse than in the original inoculum, highlighting the adaptability and robustness of the Gn pig model in studying the human gut microbiota. The observed shift from *Proteobacteria* to *Firmicutes* in Gn piglets inoculated with stunted toddler feces suggests a potential adaptation or maturation of the gut microbiota similar to the natural progression observed in humans from infancy to adulthood [61]. This indicates that the initial microbial environment significantly influences microbial community composition post-transplantation and the stability of gut microbiota in a new host [29]. Overall, we observed that the microbiome from a stunted toddler is more transferable to Gn pigs than the microbiome from a healthy toddler. This may likely result from the simplified microbial community in stunted individuals. The simplicity allows the microbiota to adapt and establish more easily in sterile Gn pigs compared to the complex, competitive communities from healthy microbiomes. A previous study by Qadri et al. noted that the higher rate of transferability of the stunted microbiome caused by the dysbiotic microbial communities could spread more easily within households and communities, particularly in environments with poor sanitation. Similarly, it was suggested that mothers with dysbiotic microbiomes may more readily transfer these communities to their infants (sterile) during birth and breastfeeding, potentially perpetuating a cycle of malnutrition and poor health [62]. These findings enhance our understanding of how malnourished microbiota perpetuate nutrient deficiencies and may help in the design of therapies like microbiota-directed foods or probiotics to counteract these effects [63, 64]. Thus, the Gn pigs colonized with stunted microbiota may serve as valuable models for studying gut microbiome impacts on growth and health. However, it is important to note that, while a malnourished microbiome may transfer more easily to Gn pigs, it may not necessarily be beneficial. Such microbiota could have adverse health effects on the recipient. Further investigation is needed to clarify these impacts and assess potential therapeutic uses of this knowledge.

Previously, using humanized Gn mice, researchers observed that microbiota of malnourished children could be partially restored with nutritional interventions, indicating a shift in bacterial communities [65–67]. Multiple studies using Gn zebrafish models have also demonstrated that diet and host genetics significantly affect microbiota composition and colonization patterns [30]. Consistent with our findings which used Gn pigs fed ultra heat temperature pasteurized bovine milk and toddlers fed breast milk, a study evaluating longitudinal establishment of human fecal microbiota in germ-free pig and mouse models revealed that 28% of taxa from humans failed to colonize either model [68], underscoring the need to exercise caution in generalizing findings from HFM transplanted animals to humans. Taken together, we tracked the microbial colonization over time in the early stages of life, providing insights into the dynamics of microbiota establishment and stability, which is crucial for understanding the development and persistence of microbiota-related health outcomes. Moreover, understanding colonization dynamics is critical when developing strategies to manipulate the gut microbiota for health benefits, such as through probiotics, prebiotics, diet or fecal microbiota transplantation. Nevertheless, we investigated the dynamics of the HFM transplantation and demonstrated that the distinct compositional and functional features of the HMT transplants persisted throughout the experiment duration. Further research focusing on genome-centric approaches and authentic toddlers’ diets could identify factors that facilitate successful colonization and the colonization profiles.

### Virulence factors and AMR in the healthy/stunted toddlers and HFM transplanted Gn pigs

Gut microbiota virulence factors and antimicrobial resistance (AMR) differ significantly between healthy and stunted individuals, contributing to varying levels of disease susceptibility and outcomes. Virulence factors in healthy individuals are typically low because the microbiota is well-regulated compared to malnourished individuals [2, 69]. However, our analysis revealed that healthy toddlers had more virulence-associated genes compared to stunted toddlers. The presence of more bacterial virulence factors in clinically healthy toddlers may indicate a more robust immune system capable of managing potential pathogens. Even though stunted toddlers, there was an increased abundance of pathobionts such as *Klebsiella*, which often express higher levels of virulence factors, we observed a lower overall abundance of virulence factors enriched in these toddlers. It is important to note that virulence factors in gut microbiota are part of a balanced microbial ecosystem that also reflects the presence of opportunistic pathogens and not necessarily linked to pathogenicity in healthy hosts [70, 71]. These unexpected and novel findings require further investigation. Conversely, stunted toddlers had a higher number of antimicrobial-resistant (AMR) bacterial genes compared to healthy ones. Mechanism of AMR in infants includes increased horizontal gene transfer, biofilm formation and stress response in bacteria. The lower prevalence of AMR genes in healthy/normal toddlers observed in this study could be due to increased beneficial bacteria and strong host immunity limiting the spread of resistant pathogens and also fewer antibiotic treatments. In contrast, the higher AMR gene content in stunted toddlers could result from more frequent infections and/or intensive repeated antibiotic treatments which contribute to the selection and persistence of resistant strains [72–75]. Moreover, malnutrition exacerbates dysbiosis by weakening the immune system and gut barrier, providing more opportunities for resistant bacteria to proliferate. Environmental factors in low resource settings such as contaminated water and food sources that harbor resistant pathogens increase the risk of acquiring AMR bacteria. This finding is significant, as it underscores the potential risks of malnutrition and/or antibiotic overuse/misuse and its impact on microbiota and resistance patterns [76, 77]. Luchen et al. in their recent review highlighted the issue of increased AMR genes in gut microbiota due to antibiotic use, in populations with frequent antibiotic exposure, leading to a disrupted microbiota and increased resistance [78]. Additionally, a recent study demonstrated that antibiotic use during the first 6 months of life is associated with an increased risk of stunting [79]. Our findings are consistent with these observations, demonstrating the complex interplay between microbial virulence factors and antimicrobial resistance in the context of malnutrition. In contrast, AMR results from the HFM Gn pigs transplanted with healthy/normal microbiota had increased enriched AMR genes compared to those transplanted with stunted toddler fecal microbiota. The decrease in AMR genes in Gn pigs transplanted with gut microbiota from stunted toddlers, (that had increased AMR genes) can be attributed to a combination of environmental changes, diet, nutritional status, lack of selective antibiotic pressure, shifts in microbiota composition, and differences in host physiology. The Gn pigs offer a drastically different environment from that of stunted toddlers, which may not favor the survival or proliferation of certain AMR-carrying bacteria, leading to a reduced abundance of these genes. On the other hand, increased AMR gene abundance in Gn pigs transplanted with healthy/normal gut microbiota may be due to factors such as enhanced horizontal gene transfer, reduced competition, stress responses in the gnotobiotic environment, and selective proliferation of resistant bacteria. Although the toddlers are healthy, their microbiota still carries a reservoir of AMR genes, which may become more prominent when transplanted into the unique/sterile environment of the Gn pig gut. Overall, these data are essential when developing strategies to mitigate AMR and understanding the potential risks posed by pathogenic AMR bacteria.

### Functional features of gut microbiota in the healthy/stunted toddlers and the HFM transplanted Gn pigs

Assessment of functional features is essential in understanding the complex interactions between gut microbes and their host and provide insights into how the microbiota influences host health and microbial diversity. This study highlighted functional similarities and differences in the gut microbiota of healthy vs. stunted toddlers, where gut microbiota of stunted toddlers exhibited an enrichment of CAZymes related to carbohydrate biosynthesis and metabolic pathways related to protein/amino acid, carbohydrate and fat catabolism. This highlights a dual microbial strategy to adapt to a resource-limited and dysfunctional gut environment (adaptive flexibility). Biosynthesis supports bacterial persistence and structural integrity, while catabolism enables bacteria to scavenge energy and nutrients from limited resources. Although this dual microbial behavior ensures bacterial survival, it often exacerbates malnutrition by depriving the host of nutrients and beneficial metabolites (e.g., SCFAs), further driving growth faltering (stunting). On the other hand, gut microbiota of healthy toddlers was enriched in CAZymes associated with carbohydrate degradation and metabolic pathways associated with anabolism, reflecting a cooperative and efficient microbiome-host relationship. Carbohydrate degradation leads to formation of SCFAs, which serve as energy sources for both bacteria and the host, while anabolism synthesizes new bacterial biomass, reflecting nutrient abundance and a focus on growth and replication. This symbiotic relationship promotes efficient energy utilization, supports host growth and metabolism, and ensures a stable, resilient gut microbiome. These findings align with the understanding that gut microbiota in healthy individuals supports a balance between anabolic and catabolic processes allowing the microbiota to efficiently utilize dietary nutrients and maintain overall gut health, whereas in malnourished individuals due to reduced nutrient availability, catabolic processes and impaired anabolic activity may dominate [17, 50, 80]. The shift towards enriched catabolic processes in stunted toddlers corroborates findings that malnutrition can result in a less diverse gut microbiota inefficient in nutrient utilization, potentially worsening nutritional deficiencies and gut dysfunction [81, 82]. We hypothesize that the enrichment of bacterial functional features supporting catabolic processes in the gut of malnourished toddlers was to efficiently utilize the little nutrients available. The metabolic imbalance observed in the stunted toddler may result in a vicious cycle, where poor nutrient absorption and inflammation further damage gut function, perpetuating malnutrition and related health challenges. Blanton et al. demonstrated that nutritional interventions could shift gut microbiota functions from biosynthesis to degradation pathways, highlighting the plasticity of the microbiota in response to diet [56]. The HFM transplanted Gn pig model mirrored many of these functional characteristics, reinforcing its utility in studying human gut microbiota. Additionally, our model demonstrated the stability of the functional characteristics of the transplanted gut microbiome. Overall, this holistic approach helps in understanding the metabolic and physiological implications of microbial community changes in toddlers. The limited number of donors and the extended freezing period of the inoculum in this study preclude robust conclusions about the influence of donor microbiota diversity on colonization success. However, this model enhances our understanding of the role of microbiota in various conditions and may aid in developing targeted microbiome-based therapies [24, 25]. The functional disparities observed in this study underline the critical role of diet and host health in shaping gut microbial ecology. Conducting future research with a wider variety of donors and authentic toddler diets will offer deeper insights into the effects of donor microbiota diversity and diet on colonization in animal models.

### Limitations

Although we did not evaluate how storage conditions affected the microbiome composition, we believe that using standardized stocks of clinically healthy/normal and malnourished/stunted toddler fecal microbiomes outweighs concerns about the potential impact of storage on the microbial populations, as previous studies have reported that frozen and fresh fecal materials are both effective in HFM transplants [83–85]. Our animals were fed on 100% ultra-high temperature pasteurized bovine milk (Parmalat) due to lack of authentic stunted/normal toddler diets. Additionally, since our Gn pigs were housed in microbiological isolators for the duration of the experiment, extending the experiment beyond 4 weeks was not feasible; therefore, the relatively short duration of the pig experiments may not have captured the long-term effects and stability of the microbiota. Extending the duration of such studies would provide a more comprehensive understanding of microbiota changes over time and their long-term impact on health.

Although our robust and physiologically relevant model recapitulates the stunting-associated microbial signatures and their impact on host health, several limitations exist. Those include: the absence of small intestinal microbiota or histological analyses limits direct conclusions about nutrient absorption and mucosal immunity. Moreover, the absence of solid food similar to participant diets may have biased colonization trajectories. Additionally, the small number of human donor samples, while constrained by the high cost of Gn piglet studies, limits the statistical power of microbiota comparisons. Thus, the current findings should be interpreted as hypothesis-generating, not confirmatory. These issues will be prioritized in follow-up experiments.

## Conclusions

To our knowledge, this is the first attempt of comparative/simultaneous transfer of normal vs. stunted toddlers’ microbiota in a Gn pig model. This study highlights similarities and significant differences in fecal microbiota composition, diversity, and function between clinically healthy/normal and malnourished/stunted toddlers which is vital for developing targeted interventions for malnourished populations, consequently advancing microbiome-based diagnosis and personalized medicine. The HFM transplanted Gn pig model allowed us to characterize and compare the microbial community composition post-transplantation and functional features of the human fecal microbiota from stunted versus healthy toddlers, thus providing a valuable tool for studying the impact of malnutrition on gastrointestinal health. Moreover, the use of Gn pigs as a model is innovative, since most prior research focused on static snapshots of microbiota composition, our longitudinal design offers insights into how these microbial communities establish and evolve over time in a controlled environment (germ-free conditions). The identification of specific bacterial taxa significantly enriched in each cohort, could pinpoint potential microbial biomarkers or therapeutic targets, which is a step forward from previous research that might have only noted general differences in diversity or broad taxonomic groups. The increased rate of transferability observed in the malnourished/stunted microbiome suggests that dysbiotic microbial communities could spread more easily within households and communities, particularly in environments with poor sanitation. Besides, our study provide a proof-of-concept that malnutrition-associated physiological alterations start early in childhood, and mothers with dysbiotic microbiomes may readily transfer these communities to their infants during birth and breastfeeding, potentially perpetuating a cycle of malnutrition and poor health. By examining the functional and compositional similarities and differences in the fecal microbiota of malnourished versus clinically healthy/normal toddlers, this study contributes to our understanding of how nutritional status influences gut health and overall well-being in early stages of childhood. Our novel findings on the diversity and maintenance of AMR and virulence bacterial genes indicate a need for further research and further highlight the intricate host-microbiome interconnections in the development of childhood malnutrition and stunting. While conclusions about human donor differences must be viewed cautiously due to limited sample size, our findings provide a foundation for future studies linking gut microbial features to growth, nutrient utilization, and immune development. Future research in this area could lead to the development of microbiota-targeted therapies combined with nutritional interventions to alleviate the adverse effects of malnutrition and promote optimal growth and development in vulnerable populations.

## Electronic supplementary material

Below is the link to the electronic supplementary material.

Supplementary Material 1:
Fig. S1: (a) Box and whiskers plot for estimated alpha diversity indices Shannon index) and (b) principal coordinate analysis (PCOA) showing beta diversity of the bacterial species present in the feces of toddlers (normal/stunted).
Fig. S2: Stacked bar plots of relative abundance of the phyla (a), families (b), top 20 genera (c) and top 20 species (d) of bacteria in feces of normal and stunted toddlers. NB: sample 6_CP04824 was grouped as stunted toddler but has bacterial composition and abundance similar to normal toddlers, hence was excluded for further comparative analysis.
Fig. S3: (a) Ven diagram showing unique/shared bacterial genera between normal (n=3) and stunted (n=2) toddlers. (b) Histograms illustrating significantly different enriched bacterial species between normal and stunted toddlers in LDA Effective Size (LEfSe) analysis (LDA>2.5, p<0.05).
Fig. S4: (a) heatmap showing mean relative abundance of bacterial virulence factors among the toddler groups. (b) Histogram illustrating significantly different enriched bacterial virulence genes among the toddlers in LDA Effective Size (LEfSe) analysis. (white color indicates the bacteria species was absent in the fecal sample).
Fig. S5: (a) Heatmap showing relative abundance of AMR bacterial classes among the toddler groups. (b) Histogram illustrating significantly different enriched bacterial AMR genes among the toddlers in LDA Effective Size (LEfSe) analysis. (* p<0.5, *** p<0.001).
Fig. S6: Histograms illustrating significantly different enriched bacterial AMR genes among the toddlers and HFM-Gn pigs cohorts in LDA Effective Size (LEfSe) analysis.
Fig. S7: (a) Bar graph showing the mean relative abundance of CAZymes among the toddler groups. (b) Histograms illustrating significantly different enriched bacterial metabolic pathways among the toddlers in LDA Effective Size (LEfSe) analysis. Glycoside hydrolases (GHs), glycosyltransferases (GTs, carbohydrate-binding modules (CBMs) and Auxiliary Activities (AAs). Human_N = Normal/healthy, Human_S = Stunted/malnourished.
Fig. S8: Heat map showing relative abundance of top 20 metabolic pathways among the toddlers and Gn pig cohorts.
Fig. S9: Heat map showing relative abundance of top 20 enzyme commissions among the toddlers and Gn pigs cohorts.
Fig. S10: Histograms illustrating significantly different enriched enzyme commissions among the cohorts in LDA Effective Size (LEfSe) analysis (LDA>2.5; p<0.05).
Fig. S11: Heat map showing relative abundance of top 20 protein families among the cohorts.
Fig. S12: Histograms illustrating significantly different enriched protein families (pfam) among the cohorts in LDA Effective Size (LEfSe) analysis (LDA>2.5; p<0.05).
Fig. S13: Heat map showing relative abundance of top 20 GO terms among the cohorts.
Fig. S14: Histograms illustrating significantly different enriched GO terms among the cohorts in LDA Effective Size analysis.

## Data Availability

The authors confirm that the data supporting the findings of this study are available within the article [and/or] its supplementary materials. The metagenomics data that support the findings of this study have been deposited in the NCBI SRA database, reference number PRJNA1228785 (http://www.ncbi.nlm.nih.gov/bioproject/1228785).
