# Supplementary material for: Stunting is associated with persistent and transferable alterations in the gut microbiome
Source: Gut Pathog. 2025 Jun 25;17:49. doi: 10.1186/s13099-025-00723-2 (PMC12199511; doi:10.1186/s13099-025-00723-2)
Supplement: Supplementary file 1 — Supplementary Material 1: Fig. S1: (a) Box and whiskers plot for estimated alpha diversity indices Shannon index) and (b) principal coordinate analysis (PCOA) showing beta diversity of the bacterial species present in the feces of toddlers (normal/stunted). Fig. S2: Stacked bar plots of relative abundance of the phyla (a), families (b), top 20 genera (c) and top 20 species (d) of bacteria in feces of normal and stunted toddlers. NB: sample 6_CP04824 was grouped as stunted toddler but has bacterial composition and abundance similar to normal toddlers, hence was excluded for further comparative analysis. Fig. S3: (a) Ven diagram showing unique/shared bacterial genera between normal (n=3) and stunted (n=2) toddlers. (b) Histograms illustrating significantly different enriched bacterial species between normal and stunted toddlers in LDA Effective Size (LEfSe) analysis (LDA>2.5, p<0.05). Fig. S4: (a) heatmap showing mean relative abundance of bacterial virulence factors among the toddler groups. (b) Histogram illustrating significantly different enriched bacterial virulence genes among the toddlers in LDA Effective Size (LEfSe) analysis. (white color indicates the bacteria species was absent in the fecal sample). Fig. S5: (a) Heatmap showing relative abundance of AMR bacterial classes among the toddler groups. (b) Histogram illustrating significantly different enriched bacterial AMR genes among the toddlers in LDA Effective Size (LEfSe) analysis. (* p<0.5, *** p<0.001). Fig. S6: Histograms illustrating significantly different enriched bacterial AMR genes among the toddlers and HFM-Gn pigs cohorts in LDA Effective Size (LEfSe) analysis. Fig. S7: (a) Bar graph showing the mean relative abundance of CAZymes among the toddler groups. (b) Histograms illustrating significantly different enriched bacterial metabolic pathways among the toddlers in LDA Effective Size (LEfSe) analysis. Glycoside hydrolases (GHs), glycosyltransferases (GTs, carbohydrate-binding modules (CBMs) and A [file 13099_2025_723_MOESM1_ESM.docx]

**Stunting is associated with persistent and transferable alterations in the gut microbiome.**

**Supplementary Figures**

**
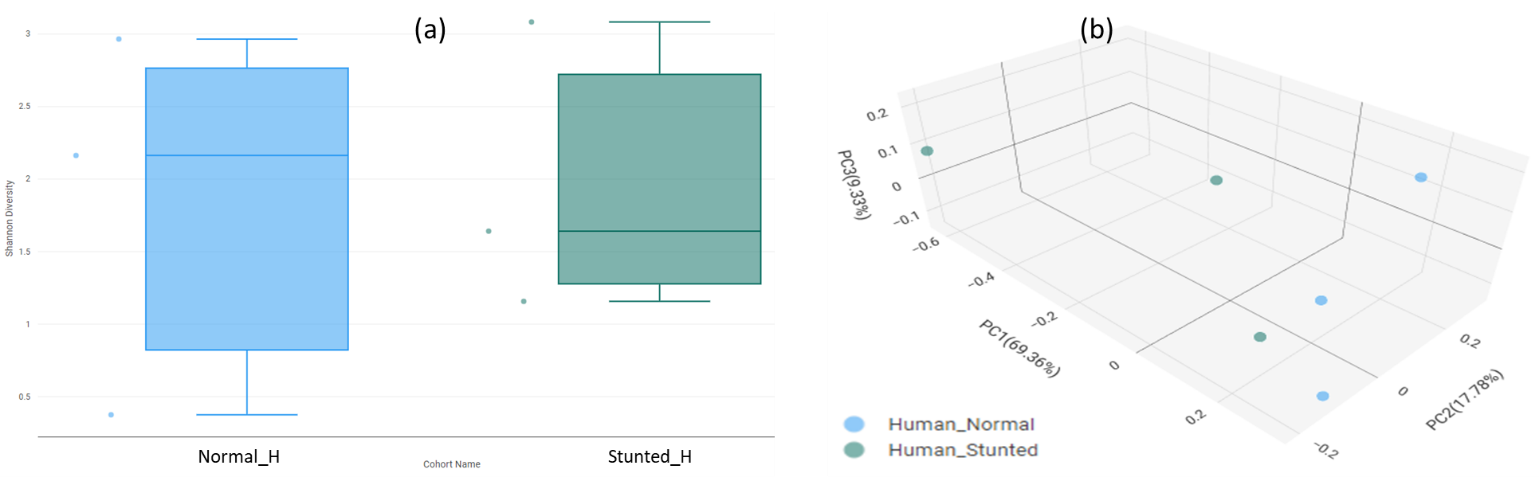
**

**Fig. S1:** (a) Box and whiskers plot for estimated alpha diversity indices Shannon index) and (b) principal coordinate analysis (PCOA) showing beta diversity of the bacterial species present in the feces of toddlers (normal/stunted).


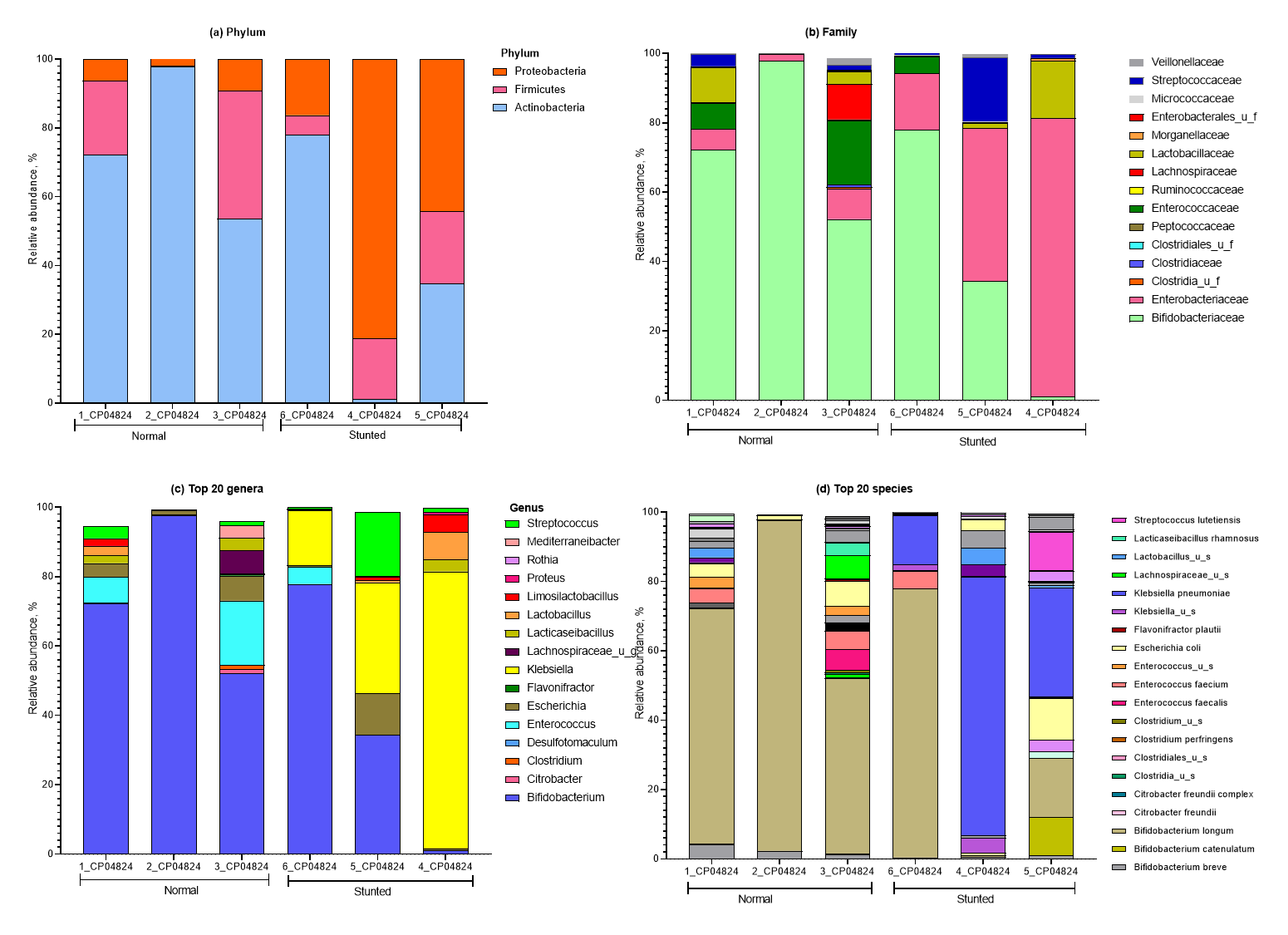


**Fig. S2:** Stacked bar plots of relative abundance of the phyla (a), families (b), top 20 genera (c) and top 20 species (d) of bacteria in feces of normal and stunted toddlers. NB: sample 6_CP04824 was grouped as stunted toddler but has bacterial composition and abundance similar to normal toddlers, hence was excluded for further comparative analysis.


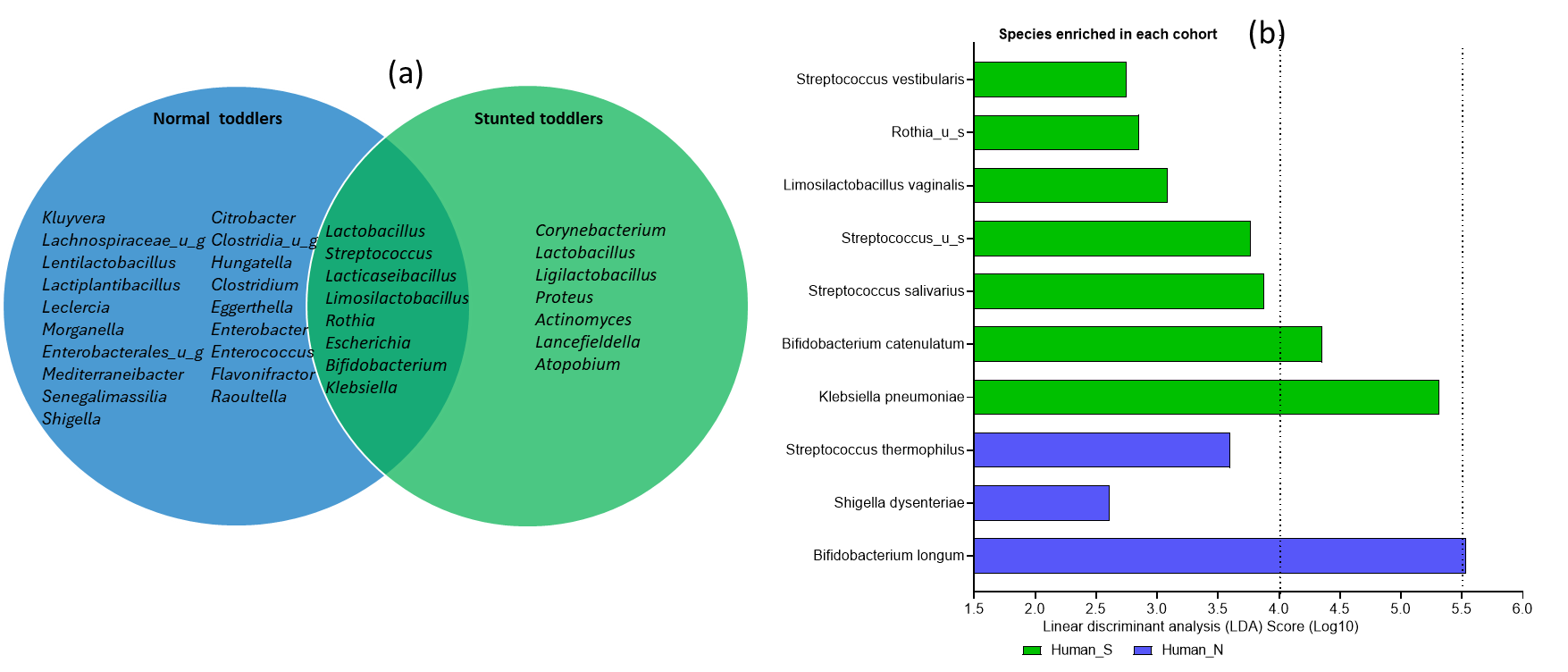


**Fig. S3:** (a) Ven diagram showing unique/shared bacterial genera between normal (n=3) and stunted (n=2) toddlers. (b) Histograms illustrating significantly different enriched bacterial species between normal and stunted toddlers in LDA Effective Size (LEfSe) analysis (LDA>2.5, p<0.05).


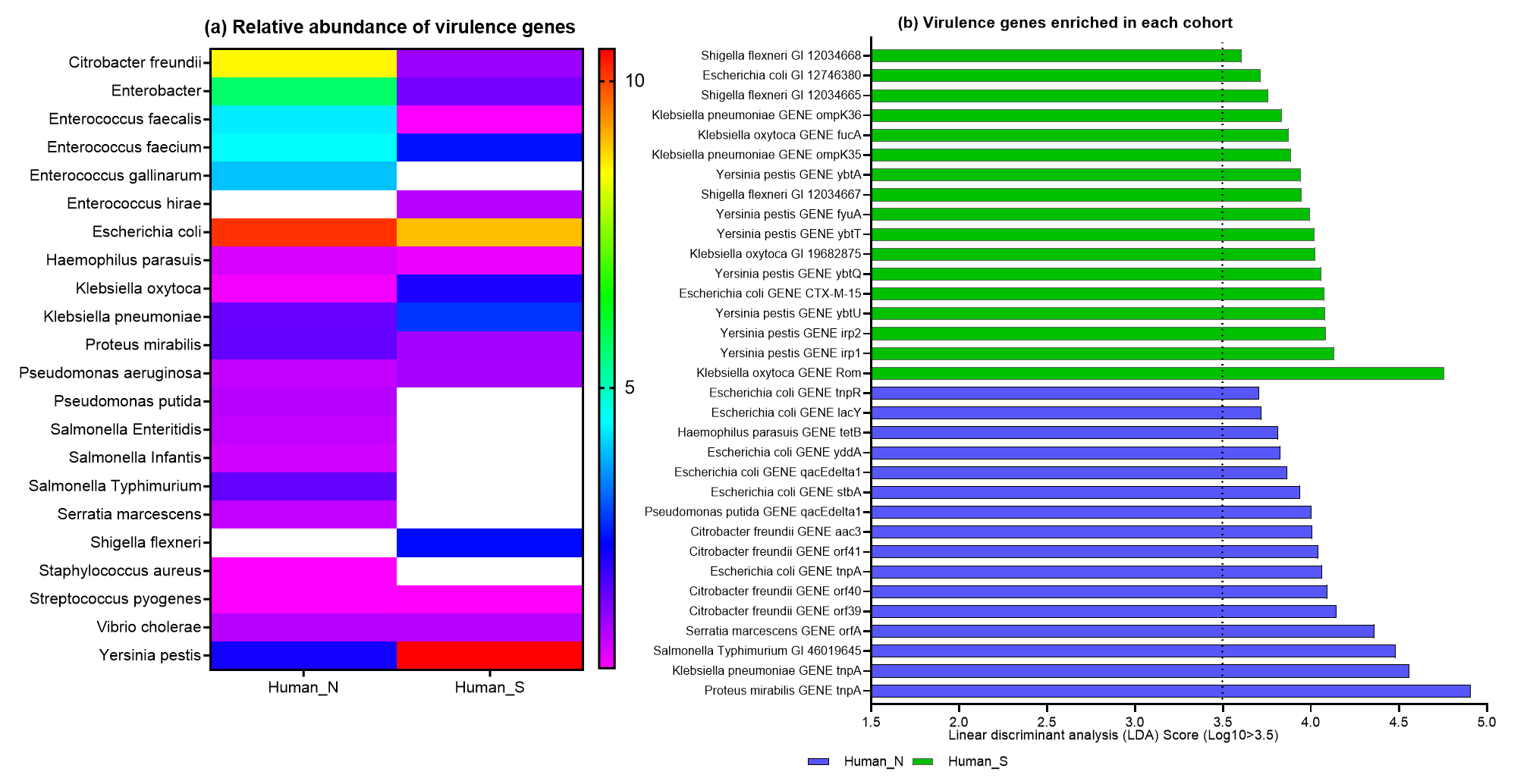


**Fig. S4**: (a) heatmap showing mean relative abundance of bacterial virulence factors among the toddler groups. (b) Histogram illustrating significantly different enriched bacterial virulence genes among the toddlers in LDA Effective Size (LEfSe) analysis. (white color indicates the bacteria species was absent in the fecal sample)


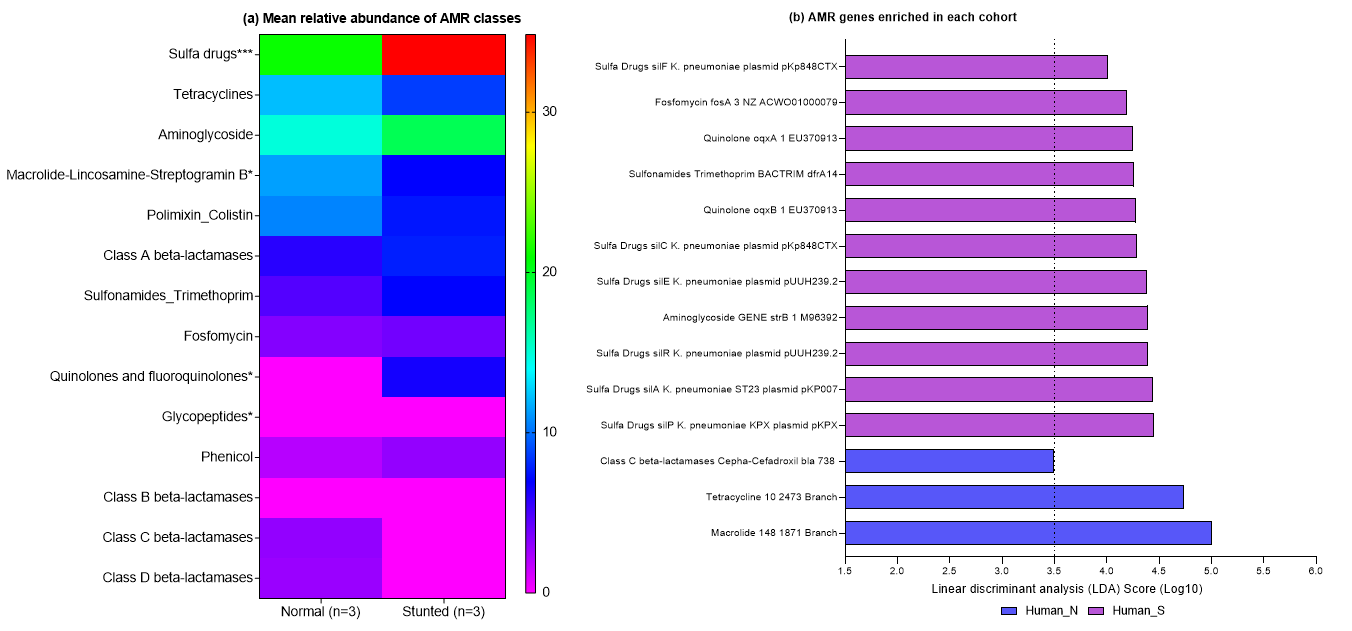


**Fig. S5:** (a) Heatmap showing relative abundance of AMR bacterial classes among the toddler groups. (b) Histogram illustrating significantly different enriched bacterial AMR genes among the toddlers in LDA Effective Size (LEfSe) analysis. (* p<0.5, *** p<0.001)


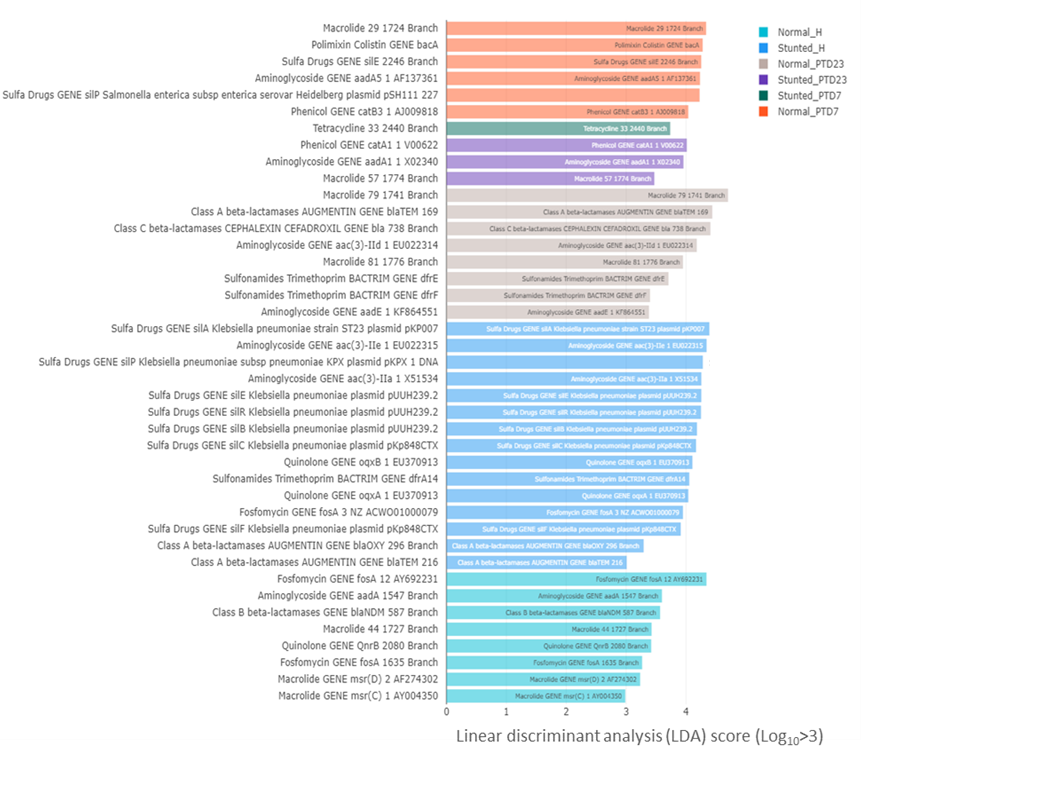


**Fig. S6**: Histograms illustrating significantly different enriched bacterial AMR genes among the toddlers and HFM-Gn pigs cohorts in LDA Effective Size (LEfSe) analysis.


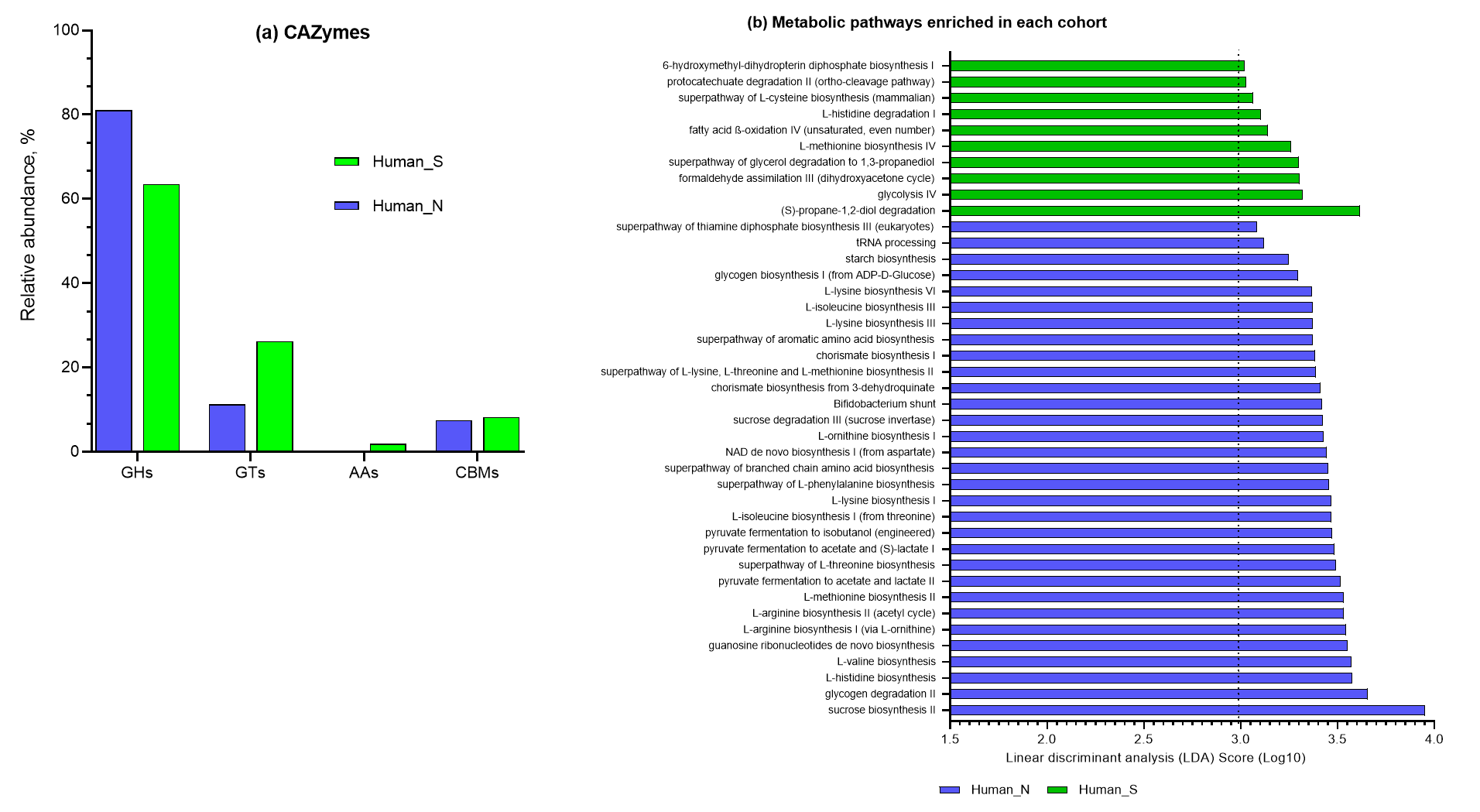


**Fig. S7**: (a) Bar graph showing the mean relative abundance of CAZymes among the toddler groups. (b) Histograms illustrating significantly different enriched bacterial metabolic pathways among the toddlers in LDA Effective Size (LEfSe) analysis. Glycoside hydrolases (GHs), glycosyltransferases (GTs, carbohydrate-binding modules (CBMs) and Auxiliary Activities (AAs). Human_N = Normal/healthy, Human_S = Stunted/malnourished.


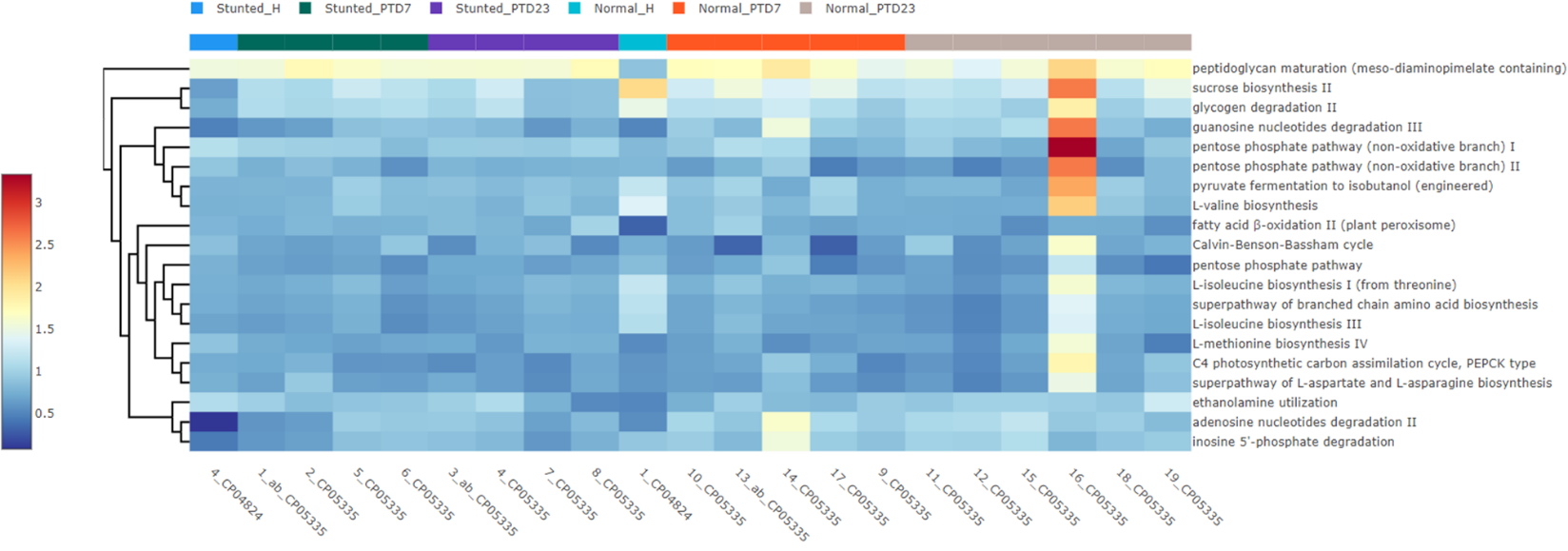


**Fig. S8**: Heat map showing relative abundance of top 20 metabolic pathways among the toddlers and Gn pig cohorts


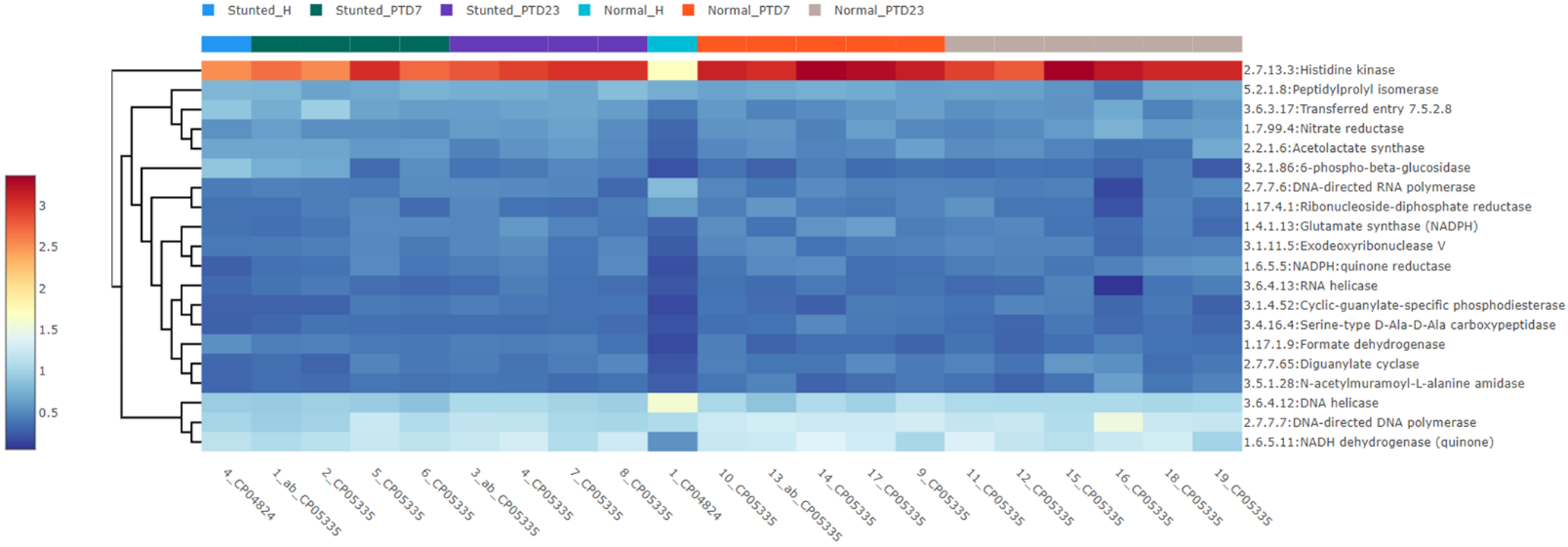


**Fig. S9**: Heat map showing relative abundance of top 20 enzyme commissions among the toddlers and Gn pigs cohorts


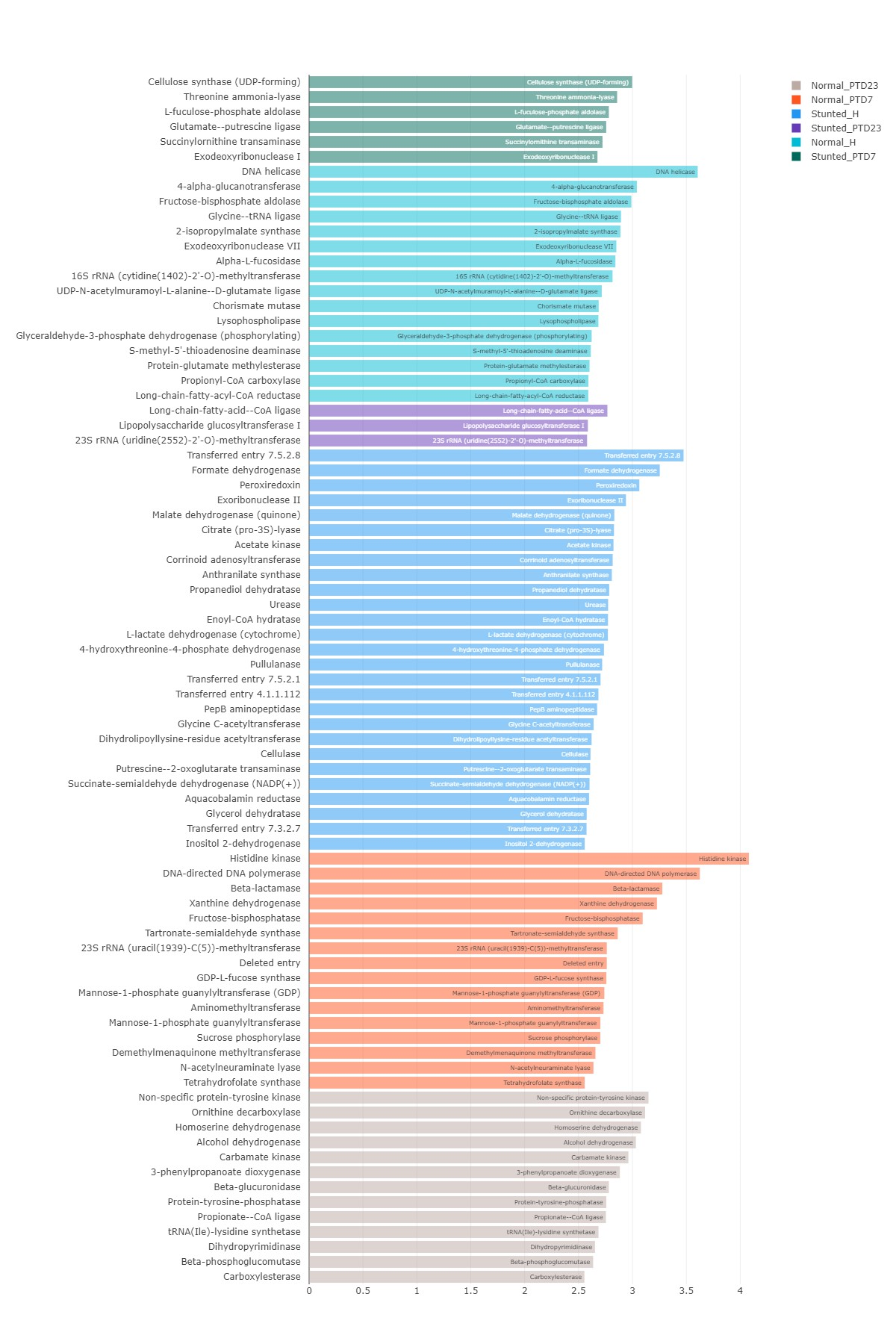


**Fig. S10**: Histograms illustrating significantly different enriched enzyme commissions among the cohorts in LDA Effective Size (LEfSe) analysis (LDA>2.5; p<0.05).


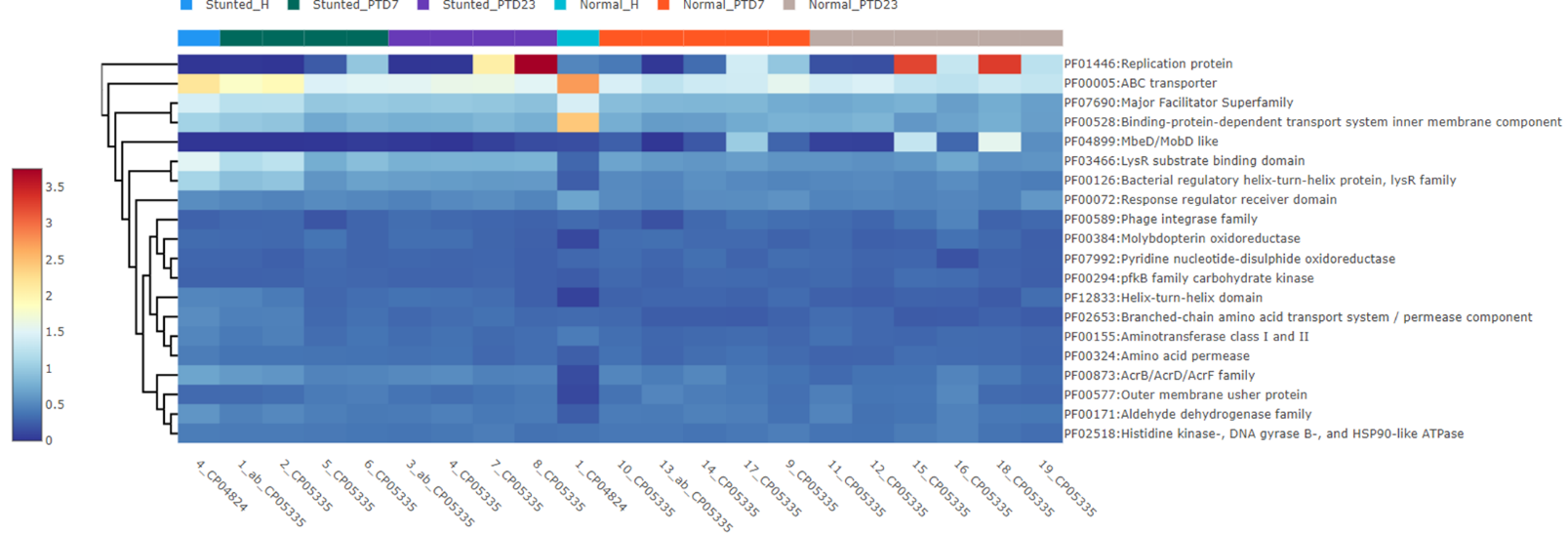


**Fig. S11**: Heat map showing relative abundance of top 20 protein families among the cohorts


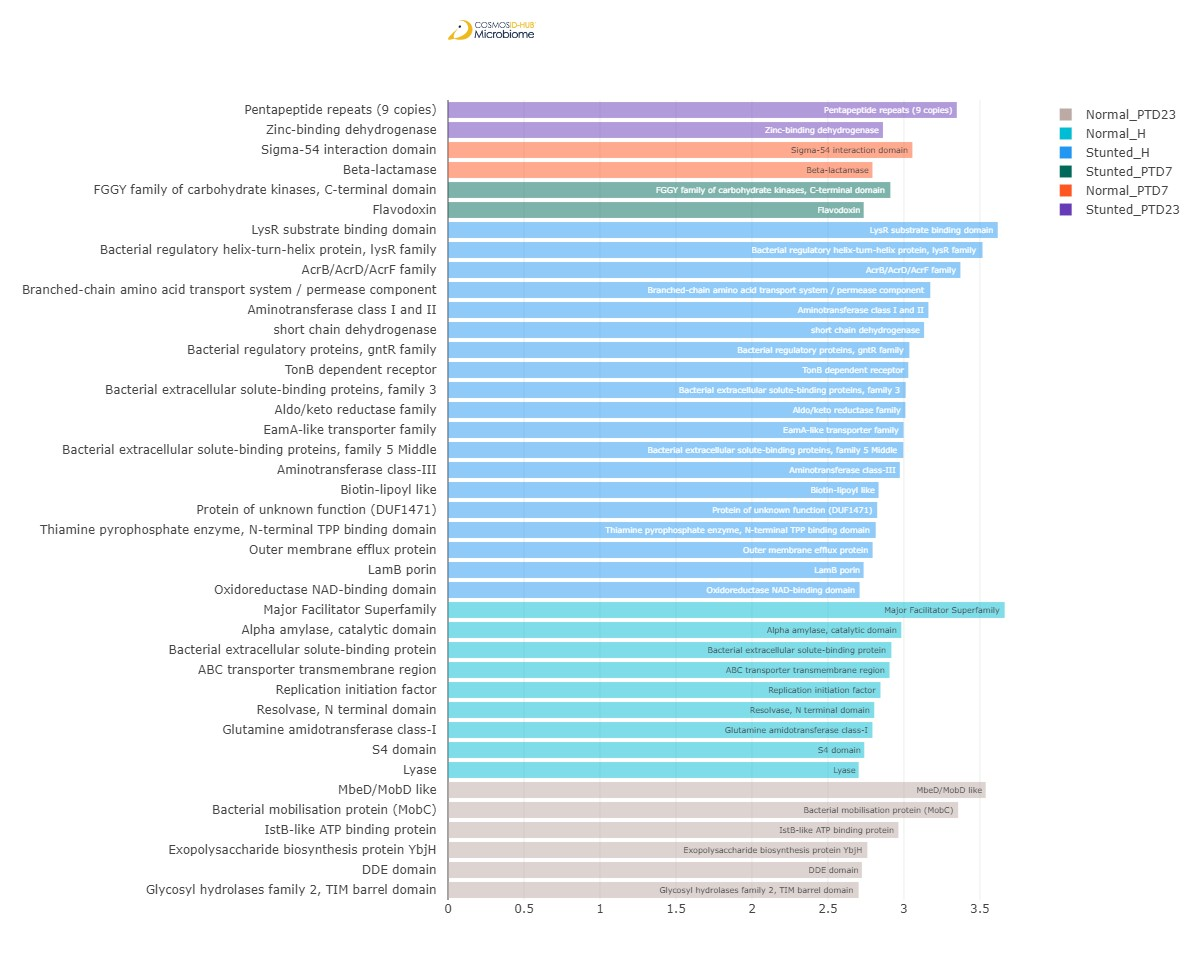


**Fig. S12**: Histograms illustrating significantly different enriched protein families (pfam) among the cohorts in LDA Effective Size (LEfSe) analysis (LDA>2.5; p<0.05).


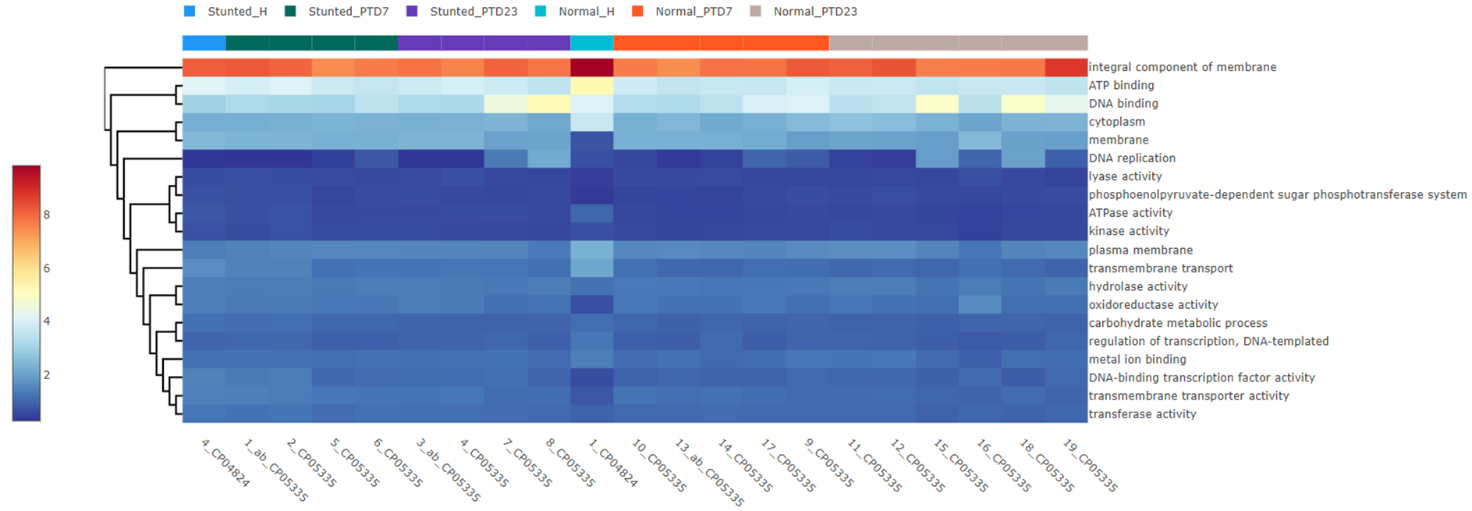


**Fig. S13**: Heat map showing relative abundance of top 20 GO terms among the cohorts


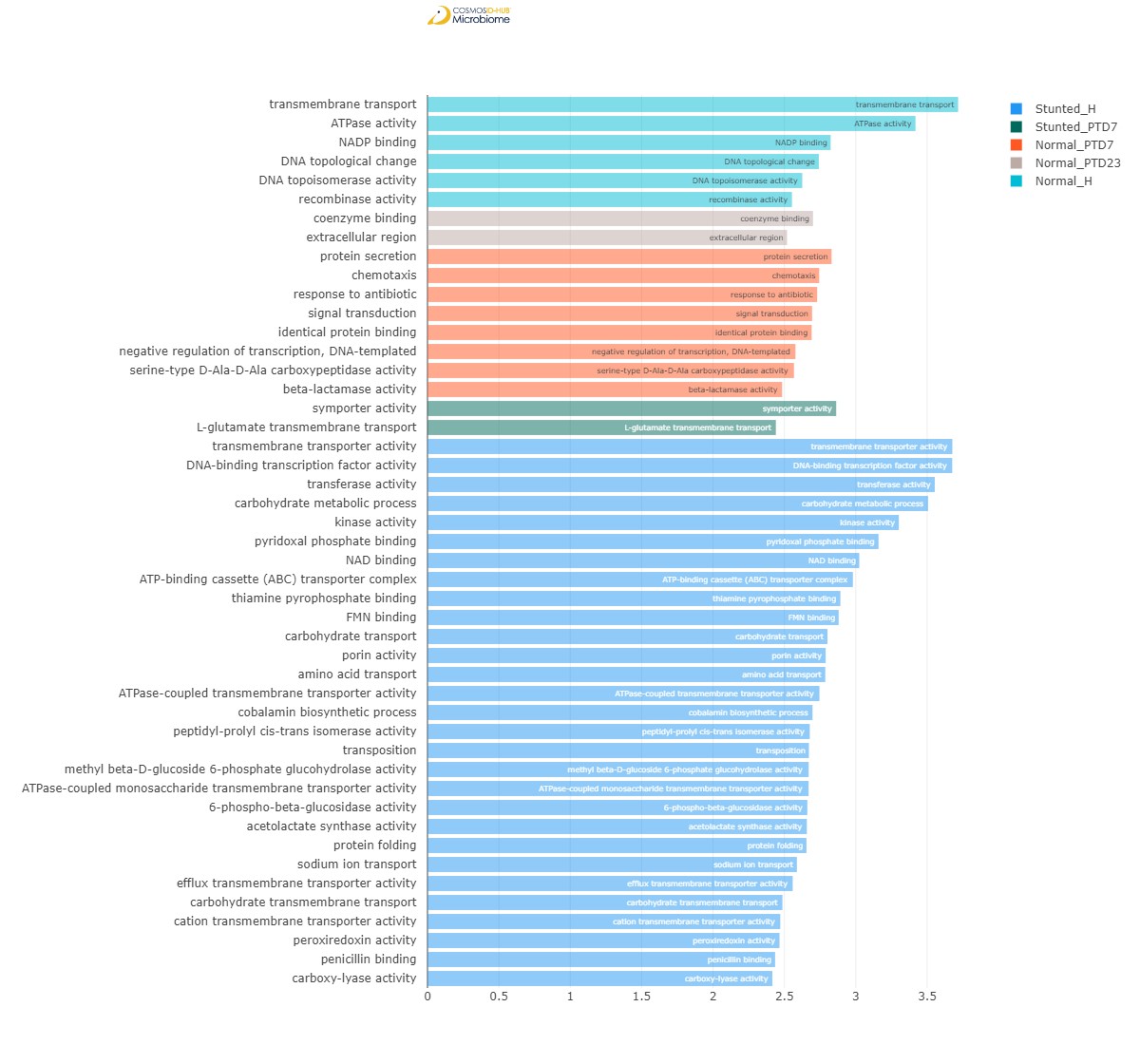


**Fig. S14**: Histograms illustrating significantly different enriched GO terms among the cohorts in LDA Effective Size analysis.
